# Supplementary material for: Constrained Allocation Flux Balance Analysis
Source: PLoS Comput Biol. 2016 Jun 29;12(6):e1004913. doi: 10.1371/journal.pcbi.1004913 (PMC4927118; doi:10.1371/journal.pcbi.1004913)
Supplement: S1 Text — Supplementary notes, tables, figures and references. (PDF) [file pcbi.1004913.s001.pdf]

Constrained Allocation Flux Balance Analysis

Supplementary Notes

M. Mori, T. Hwa, O.C. Martin, A. De Martino, E. Marinari

Contents

Note A. The linear enzyme–flux relation **2**

Note B. The choice of the control parameter **3**

Note C. Extension to different growth media and/or bacterial species **5**
CAFBA for E. coli growth in a generic medium with a set of limiting nutrients 5
Application to different *E. coli* strains or bacterial species 5

Note D. Translational inhibition and protein over–expression in CAFBA solutions **7**
Growth rate 7
Proteome fractions 8
Fluxes and Q–limitation 9

Note E. Case of inhomogeneous proteome costs **11**
Sample to sample fluctuations and fluctuations in the weights 11

Note F. Growth-dependent biomass composition **14**
Implementation of a growth-dependent biomass composition in CAFBA 15

Supplementary Tables **20**

Supplementary Figures **21**

Supplementary References **32**

Note A. The linear enzyme–flux relation

One of our basic assumptions is the following flux–enzyme relation:

$$\phi_i = \phi_{i,0} + w_i|v_i| \ . \tag{S1}$$

Eq. (S1) is our constitutive relation between proteome fractions and fluxes. Generally speaking, this relation expresses the fact that larger fluxes require a larger proteome share in order to be sustained. Here we analyze briefly its origin and detailed implementation within CAFBA. The general case is treated extensively in the Main Text, but we will repeat the derivation in this section in the specialized case of Michaelis–Menten kinetics.

Let us consider a single irreversible reaction with a flux v_i described by Michaelis-Menten kinetics. Calling $[s]$ and $[E_i]$ the substrate and enzyme concentrations, we have:

$$v_i = \frac{V}{M_{DW}} k_{cat,i}[E_i] \frac{[s]}{[s] + K_M} \quad , \tag{S2}$$

where V is the cell volume, M_{DW} is the cellular dry weight mass and K_M the Michaelis constant of the reaction. It is worth remembering that in Eq. (S2) the dilution flux $-\lambda[p]$ of the reaction product has been neglected, as usually done in steady state calculations, since the typical concentration ranges of the metabolites are such that the dilution flux is much smaller than the flux processed by the enzymes. (See however Ref. [1] for an attempt to explicity model dilution of intermediate metabolites in genome–scale networks.) The function $f([s], [p])$ introduced in the main text is given in this case by $[s]/([s] + K_M)$. Enzyme concentration and the corresponding proteome fractions are related by:

$$[E_i] = \frac{M_{TP}}{V\mu_i} \phi_i \quad , \tag{S3}$$

where M_{TP} is the total protein mass and μ_i is the enzyme molecular mass. Therefore, Eq. (S2) can be written as:

$$v_i = \kappa_{cat,i}\phi_i \frac{[s]}{[s] + K_M} \quad , \quad \text{with} \quad \kappa_{cat,i} = \frac{k_{cat,i}}{\mu_i} \frac{M_{TP}}{M_{DW}} \quad . \tag{S4}$$

If the reaction is saturating, we obtain the linear enzyme–flux relation:

$$\phi_i = \frac{1}{\kappa_{cat,i}} v_i \tag{S5}$$

This relation is Eq. (S1) with $w_i = 1/\kappa_{cat,i}$ and $\phi_{i,0} = 0$. However, this case is not very realistic, as it is known that the average saturation level in metabolic reactions varies in different growth conditions [2, 3].

We consider instead the case in which the substrate concentration is directly proportional to the flux itself, as $[s] = \alpha v_i$. As described in the main text, this is a common situation for glycolytic enzymes. In this case one obtains another linear relation between the enzyme levels and the fluxes:

$$\phi_i = \frac{K_M}{\alpha} + \frac{1}{\kappa_{cat,i}} v_i \ . \tag{S6}$$

The presence of baseline expression levels for each enzyme can be seen as the direct consequence of the presence of finite substrate concentrations. The existence of such basal level is also supported by quantitative proteomics data [4], and can be then introduced as constant offset $\phi_{i,0} > 0$.

It is interesting to express the enzyme proteome fraction as a function of substrate concentration, $[s_i]$. In this case, one obtains the simple expression:

$$\phi_i = \frac{K_M}{\alpha} + \frac{1}{\alpha\kappa_{cat,i}}[s] = \phi_{i,0}\left(1 + \frac{1}{\kappa_{cat,i}} \frac{[s]}{K_M}\right) \ . \tag{S7}$$

where $\phi_{i,0} = K_M/\alpha$. This suggests that positive regulation of the enzyme i by its own substrate may enforce the linear flux–substrate relation. This feedforward activation regulatory motif is seen in quite a number of metabolic control, e.g., the activation of lower glycolysis proteins by fructose bisphosphate (via Cra) [5], the activation of Pyruvate dehydrogenase by pyruvate via PdhR [6]; the upregulation of ribosome synthesis by amino acid (via ppGpp) also belongs to this regulatory class.

Note B. The choice of the control parameter

In standard FBA, the carbon intake flux (or, more precisely, the upper bound on the carbon intake flux) is used as a control parameter by which the growth rate can be tuned, so that, for instance, keeping all other flux specifications (upper and lower bounds, ATP maintenance, etc.) fixed one can obtain different values of λ by simply changing the glucose intake flux. Other models [7, 8] employ global constraints on fluxes as control parameters for λ, with biological rationale based e.g. on molecular crowding constraints; finally, other models use a combination of global constraints and explicit bounds on the glucose intake flux [9].

In this work control of λ is instead achieved by tuning the weight w_C, corresponding to the proteome fraction per unit flux allocated for carbon scavenging and intake. (For sakes of simplicity, we shall henceforth refer to glucose as the main carbon source.) In other words, in CAFBA the glucose intake is a free variable. The exchange fluxes of all other carbon sources have lower bound equal to 0, so that the metabolites can only be excreted. In essence, the reason for this choice is that w_C is a convenient proxy for the extracellular glucose *level*. To see this, let us assume Michaelis–Menten kinetics for the glucose transport. In this case, one can see that the proteome fraction ϕ_C of glucose transporters and the glucose flux v_C are related by

$$v_C = \kappa_{cat} \phi_C \frac{[g]}{[g] + K_M} \quad , \tag{S8}$$

where $[g]$ denotes the extracellular glucose level and κ_{cat} is a rescaled turnover, as described in the Main Text. Therefore, the proteome fraction required to sustain a glucose intake flux at least equal to v_C in presence of an extracellular glucose level $[g]$ is given by

$$\phi_C = v_C \times \frac{1}{\kappa_{cat}} \left(1 + \frac{K_M}{[g]} \right) \quad . \tag{S9}$$

Crucially, ϕ_C increases as $[g]$ is reduced, consistently with the expectation that, as the substrate levels goes down, a larger investment in terms of proteome fraction is needed to sustain the same intake flux. We can then write $\phi_C \geq w_C v_C$, with $w_C = (1 + K_M/[g])/\kappa_{cat}$. Since the relationship between w_C and $[g]$ is invertible, w_C can be used in place of $[g]$ as a control parameter: high values for w_C are associated to low substrate levels, and vice versa. Note that the largest achievable growth rate is obtained in the limit $w_C \to 0$. Fig. N1 shows how different choices of w_C affect the growth rate in CAFBA with a fixed glucose influx v_C. λ grows linearly with v_C for small intakes, reaches a maximum, and then goes to zero for large values of v_C. Once the value of w_C is given, CAFBA returns as the optimal λ the value corresponding to the maximum in those curves, and the glucose intake corresponding to it as the actual value of v_C.

Using w_C instead of v_C as a control parameter leads to striking differences in the flux configurations observed at high growth rates. In particular, in the former case (panel (A)) one observes jump discontinuities in the fluxes as w_C is varied, see Fig. N2. These sharp transitions are due to the fact that, as w_C changes, the solutions of the optimization problem display large-scale flux rearrangements that do not occur in general upon varying v_C. Such sharp transitions are usually not observed in experiments. Acetate excretion in *E. coli* starts above a critical growth (or dilution) rate [10, 11, 12]. Sigmoidal response functions are common in regulatory system, but usually they have some crossover region. Nonetheless, the biologically significant consequence is that solutions displaying carbon overflow may be found to be optimal for growth rates well below the largest achievable. On the other hand, for $w_C = 0$, when growth is modulated by changing the upper bound on the glucose intake (panel (B)), the CAFBA constraint effectively reduces to a finite capacity constraint on the enzymatic proteome sector, similar to the molecular crowding constraint that defines FBAwMC [7, 8]. In these conditions, key transitions like that related to acetate onset shift to values of λ close to the fastest achievable rates.

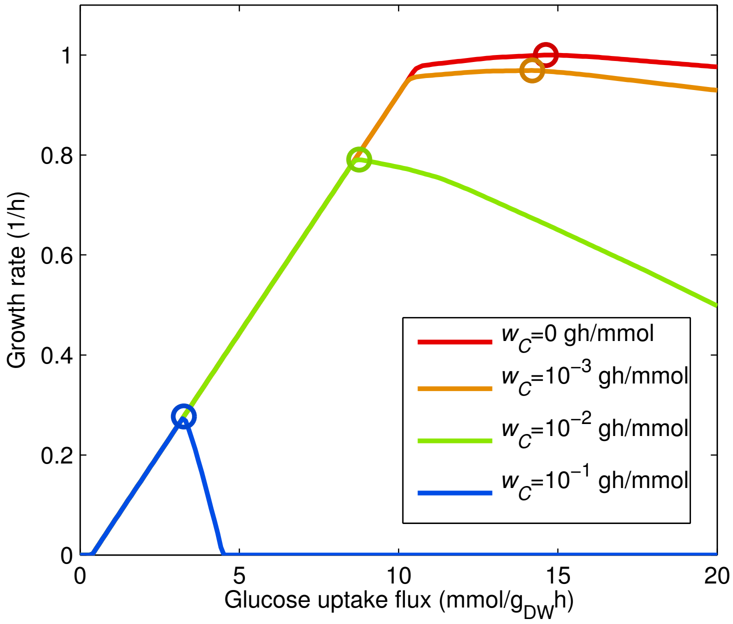

Figure N1: Growth rate of CAFBA solutions obtained as a function of the glucose uptake flux, for different degrees of carbon limitation ($w_C = 0, 10^{-3}, 10^{-2}, 10^{-1}$ $g_{DW}h/mmol$). The continuous lines show solutions obtained by directly constraining the carbon (glucose) flux; the circles indicate the CAFBA solutions when the carbon flux is not constrained.

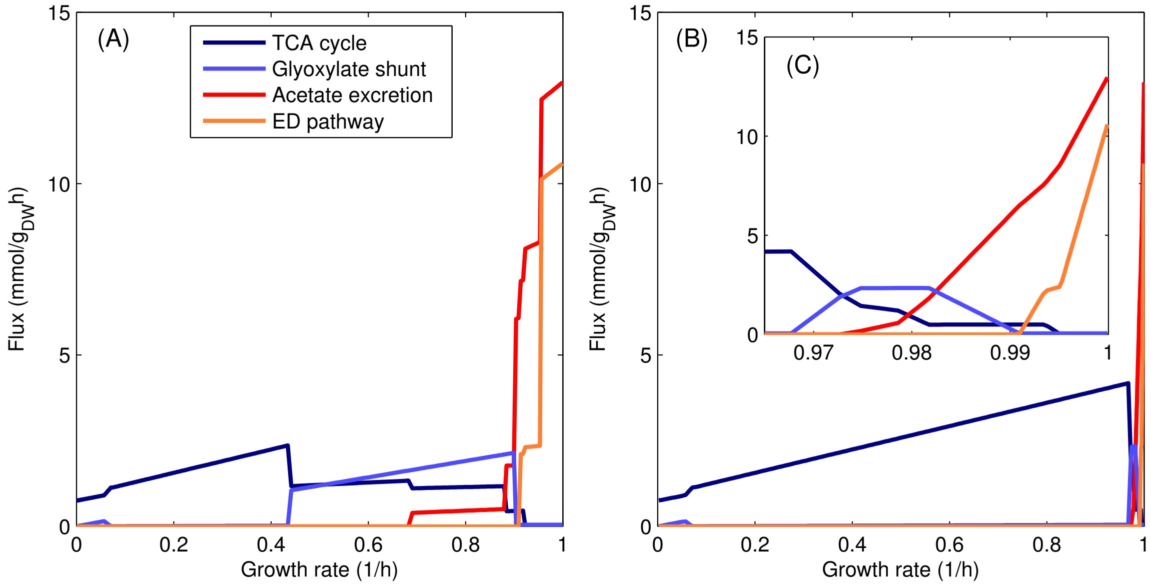

Figure N2: (**A**): CAFBA fluxes computed using w_C as the control parameter (glucose minimal medium). (**B**): CAFBA fluxes computed by imposing an upper bound on the glucose influx, at fixed $w_C = 0$. The inset (**C**) shows a detail of figure (**B**) at high growth rates.

Note C. Extension to different growth media and/or bacterial species

CAFBA for E. coli growth in a generic medium with a set of limiting nutrients

When the parameters w_R and $\phi_{\max}$, as well as the E–sector weights, are known for the strain of interest, knowledge of the substrate–specific values of the largest achievable growth rates (coming e.g. from batch culture experiments) suffices to set up CAFBA in a generic medium where different types of limitation can be analyzed. Here we describe how CAFBA can be used to study different types of limitation in *E. coli*, e.g. for growth in a rich medium or in presence of a combination of carbon sources.

The first step to set up a CAFBA (as well as standard FBA) simulation is the definition of a growth medium, that is, the list of available substrates. For each of them, the lower bound of the corresponding exchange flux v_a should be set to a large negative value, e.g. -1000 mmol/$g_{\rm DW}$h, describing an in–take. As usual, the lower bound of exchange fluxes of metabolites not available in the medium should be set to zero. Then, if one wants to study growth under limitation of substrate a, the corresponding proteome fraction $\Delta\phi_a = w_a|v_a|$ should be introduced explicitly in the CAFBA–specific proteome allocation constraint. Denoting by A the set of substrates that one is interested in limiting, the proteome allocation constraint takes the form

$$\sum_{a\in A} w_a|v_a| + \sum_{i\in E} w_i|v_i| + w_R\lambda = \phi_{\max} \ . \tag{S10}$$

The empirical substrate–specific maximum growth rate can be fixed by introducing a lower bound $w_{a,0}$ for each nutrient source (see Main Text and Supplementary Table S2 for an example). Limitation of the nutrient source a is then obtained by fixing w_a at a value $w_a > w_{a,0}$ (or by varying it so that $w_a > w_{a,0}$ always).

Example: phosphate limitation for E. coli MG1655

The results discussed in this work concern *E. coli* growth in a carbon minimal medium. Following the previous section, we describe here the detailed procedure to set up CAFBA (in the homogeneous case) for *E. coli* MG1655 strain in minimal glucose medium subject to phosphate limitation. *E. coli* is known to possess both passive and active Pi transporters, that are up-regulated in phosphate starvation [13]. Therefore, the phosphate in-take flux can be expected to depend on the extracellular phosphate level via a relation similar to the one holding for glucose, see Eq. (5) in the Main Text.

1. As described in the main text, the values of the CAFBA–specific parameters for MG1655 are $w_E = 1.55\times10^{-3}$ $g_{\rm DW}$h/mmol, $w_R = 0.169$/h and $\phi_{\max} = 48.4\%$. With these choices the maximum growth rate $\lambda_{\max}$ of the cells, obtained when all nutrient–specific weights are set to zero, is about 0.7/h.

2. The standard minimal medium for the *iJR*904 model contains H_2O, O_2, CO_2, $NH_4{}^+$, H^+, Na^+, K^+, Fe^{++}, $HPO_4{}^{--}$, $SO_4{}^{--}$, plus the carbon source (in this case, glucose). We set the lower bound on the corresponding exchange fluxes to -1000 $g_{\rm DW}$h/mmol.

3. We introduce both a glucose–specific weight w_C and an phosphate–specific weight w_P, which are both initially set to zero.

4. CAFBA solutions are computed with increasing values of w_C, until the growth rate matches the experimental growth rate in glucose minimal medium, about 0.65/h [14]. Call $w_{C,0}$ the corresponding value for the glucose weight.

5. Phosphate limitation is finally obtained by increasing w_P at fixed $w_C = w_{C,0}$.

Other types of limitation (e.g. nitrogen or oxygen) can be easily implemented by introducing the corresponding nutrient–specific weights.

Application to different *E. coli* strains or bacterial species

When applying CAFBA to other strains or species, the starting point is the corresponding contraint–based metabolic model, such as the ones available at the BiGG database [15]. The parameters w_R, $\phi_{\max}$ and the E–sector weights have then to be defined in order to set up the CAFBA proteome allocation constraint.

Slope of the R–sector The weight w_R is the slope of the R–sector of ribosome–associated proteins. For *E. coli*, the R–sector is linearly related to growth rate as $\phi_R \simeq \phi_{R,0} + w_R\lambda$. w_R is precisely the slope of the line, i.e. $w_R = \partial\phi_R/\partial\lambda$. This value can be obtained by measuring the amount of ribosomal proteins at different growth rates. In particular, w_R has been experimentally estimated to be close to 0.169 h for MG1655 strain [14] and 0.189 h for NCM3722 [16].

Fraction of allocable proteins The proteome fraction $\phi_{\max}$ corresponding to the total fraction of growth-rate dependent ("allocable") proteins. The numerical value is obtained by measuring the maximum variation of the growth rate sectors upon different kind of growth limitation. For *E. coli*, $\phi_{\max}$ has been estimated to be around 48.4% for the MG1655 strain [14] and 43% for the NCM3722 strain [16].

Definition of the E–sector. The first step is the definition of an E–sector. In CAFBA, the E–sector includes all intracellular metabolic reactions except for those relative to the carbon uptake system. As such, it does not represent a functionally homogeneous cluster of proteins that respond coherently upon modulating the growth conditions, like those identified in [4]. To each reaction i in the E–sector one should associate a weight w_i. We distinguish two cases:

Homogeneous case – All the weights w_i of the E–sector are fixed to the same value, w_E. The value of w_E has to be fixed so that the extrapolated *E. coli* growth rate in saturating carbon sources, corresponding to $w_C \to 0$, is 1/h as found in [16].

Heterogeneous case – Each w_i is sampled randomly and independently from a given probability distribution $p(w)$. The fluxes obtained as CAFBA solutions for many independent samples are then averaged. In principle, any probability distribution $p(w)$ can be chosen (e.g. $p \sim 1/w$, or a lognormal distribution). However, we find that different choices produce quantitatively similar results as long as the mean value $\langle w \rangle$ and the standard deviation $\sigma_w = \sqrt{\langle w^2 \rangle - \langle w \rangle^2}$ are similar. The latter therefore appear to be the only key parameters. Similarly to w_E in the homogeneous case, $\langle w \rangle$ is chosen so as to reproduce the growth rate in saturating glucose (1/h). σ_w, instead, has to be adjusted so as to obtain a better qualitative fit with experiments. A discussion of the results obtained with different values for the standard deviation is presented in Supplementary Note S5.

We conclude this note with some observations about the definition of the E–sector and the choice of the parameters.

First, in our application to carbon limitation, many catabolic reactions (e.g. carbon–specific uptake systems such as *lac*, *gal*, etc.) were not included into the E–sector, since they are empirically known to be upregulated in carbon limitation [4], which places them in the C–sector. Their inclusion in the E–sector does not affect results, as it simply generates an extra cost for the E–sector proportional to the carbon uptake, thereby being effectively equivalent to a rescaling of w_C.

Concerning the choice of the other parameters, we note finally that besides the nutrient–specific weights lower bounds $w_{a,0}$, the number of parameters in the homogeneous and the heterogeneous case is three for the homogeneous case (w_E, w_R and ϕ_{max}), while it is four for the heterogeneous case (the same parameters, plus the width of the E–sector fluctuations). Note, however, that one of these parameters can be arbitrarily fixed due to the homogeneity of the proteome allocation constraint, whereas increasing (resp. decreasing) w_R is roughly equivalent to decreasing (resp. increasing) $\phi_{\max}$. Therefore, the effective number of free parameters reduces to one for the homogeneous case and to two for the heterogeneous case.

Note D. Translational inhibition and protein over–expression in CAFBA solutions

The proteome allocation constraint in the homogeneous case $w_i = w_E$ reads

$$w_C v_C + w_E \sum_i |v_i| + w_R \lambda = \phi_{\max} \tag{S11}$$

where 4 parameters occur, namely w_C, w_E, w_R and $\phi_{\max} = 1 - \phi_{C,0} - \phi_{R,0} - \phi_Q$. Since w_E is fixed so as to ensure that $\lambda = \lambda_{\max} = 1/\mathrm{h}$ when $w_C = 0$, and since the above condition is invariant under a re-scaling of parameters, we are left with just two free parameters, namely w_R and $\phi_{\max}$. In the Main Text, we discuss the case in which they are fixed based on experiments [14]. Here we focus instead on how CAFBA solutions depend on them. Moreover, we study in detail how CAFBA solution allow to reproduce and extend the coarse–grained theory of proteome allocation developed in Ref. [14].

Growth rate

The Michaelis-Menten dependence of the growth rate on the quality of the nutrients is a classical finding due to Monod [17]. In the proteome partition model described in Ref. [14], the growth rate λ is found to be a Michaelis-Menten function of two parameters, namely the nutritional quality k_c and the translational capacity k_r. λ is also linearly dependent on the Q–sector proteome fraction (also incorporating overexpression of unnecessary proteins. In particular,

$$\lambda = (\phi_Q^{\max} - \phi_Q)\frac{k_c k_r}{k_c + k_r} \; . \tag{S12}$$

A similar result can be obtained through a fitting procedure in CAFBA. Identifying $w_C = 1/k_c$ and $w_R = 1/k_r$, the CAFBA-predicted growth rate turns out to be described with high accuracy by the expression

$$\lambda = \mu\big(\phi_Q^{\max} - \phi_Q\big)\frac{\left(1 - \dfrac{k_c^{\min}}{k_c}\right)}{1 + \dfrac{K_M^c}{k_c} + \dfrac{K_M^r}{k_r}} \quad , \tag{S13}$$

where μ, $k_c^{\min}$, K_M^C and K_M^R are parameters whose values are listed in Table N1. Notice that λ is a Michaelis-Menten function of k_r for fixed k_c, and reduces to a Michaelis-Menten function of k_c for fixed k_r in the $k_c^{min} \ll k_c$ limit. This approximation works well if the growth rate is not too small ($\lambda \gtrsim 0.1/h$). Furthermore, λ decreases linearly with increasing ϕ_Q. Growth rate is shown in Fig. S2 as a function of k_c and k_r, together with acetate expression.

Fit results

Parameter	$k_c \leq k_{c,ac}$ ($\lambda \leq \lambda_{ac}$)	$k_c \geq k_{c,ac}(\lambda \geq \lambda_{ac})$
μ	3.39/h	3.51 /h
$k_c^{\min}$	0.808 mmol/$\mathrm{g_{DW}}$h	1.79 mmol/$\mathrm{g_{DW}}$h
K_M^c	35.0 mmol/$\mathrm{g_{DW}}$h	42.5 mmol/$\mathrm{g_{DW}}$h
K_M^r	3.34/h	3.44/h
$\phi_Q^{\max}$	91%	91%

Table N1: Fit results, Eqs. (S14) and (S15), expressed as a function of the parameters appearing in Eq. (S13). The value of $\phi_Q^{\max}$ is obtained directly fitting growth rate as a function of ϕ_Q, see Fig. N5. The value $k_{c,ac} \sim$ 200 mmol/$\mathrm{g_{DW}}$his the value at which the acetate starts to be excreted, roughtly at $\lambda_{ac} \sim 0.8$/h. All results are obtained with $w_E = 0.00083$ and $\phi_{R,0} = 6.6\%$. Note that $\phi_Q^{\max}$ is less than $(1 - \phi_{R,0}) = 93.4\%$, due to the basal enzymatic proteome fraction ϕ_E induced by the ATP mantainence flux.

The values of the parameters in Eq. (S13) are obtained by a sequence of steps. We first assume the linear relation $k_r/\lambda = f_1(k_c) \cdot k_r + f_2(k_c)$. This relation is tested for different values of k_c, thus defining the

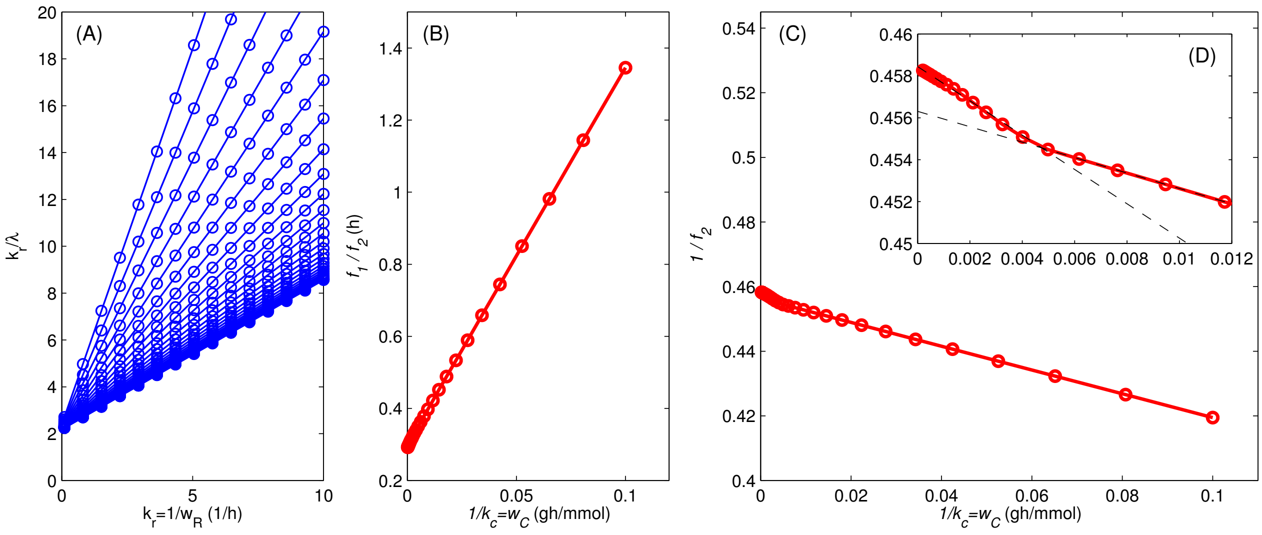

Figure N3: (**A**): k_r/λ against k_r for different values of k_c. For each k_c, k_r/λ is a linear function of k_r with slope $f_1(k_c)$ and y–intercept $f_2(k_c)$. (**B**): f_1/f_2 plotted against $1/k_c$, see Eq. (S14). (**C**): $1/f_2$ plotted against $1/k_c$, see Eq. (S15). Inset (**D**) shows a zoom on the crossover at $k_{c,ac} \sim 200$ mmol/$\mathrm{g_{DW}}$h($w_{C,ac} \sim 0.005$ $\mathrm{g_{DW}}$h/mmol). The dashed lines are linear fits performed on all points before or after the one at $w_{C,ac}$.

two functions f_1 and f_2. Then, we impose the following linear relations:

$$f_1/f_2 = c_1 + c_2 \frac{1}{k_c} \ , \tag{S14}$$

$$1/f_2 = c_3 - c_4 \frac{1}{k_c} \ . \tag{S15}$$

The minus sign in Eq. (S15) allows to consider positive definite constants $c_1, \ldots, c_4$. The parameters in Eq. (S13) are then obtained as $\mu(\phi_Q^{\max} - \phi_Q) = c_3/c_1$, $k_c^{\min} = c_4/c_3$, $K_M^c = c_2/c_1$ and $K_M^r = 1/c_1$. Finally, the prefactor μ is obtained by solving CAFBA for different values of ϕ_Q (see Fig. N5).

The fitting procedure is illustrated in Fig. N3. The results of the fit are impressively good from very low growth rates to the acetate switch growth rate, $\lambda_{ac} \sim 0.9/h$ ($k_{c,ac} \sim 200$).For $k_c \gtrsim k_{c,ac}$ the coefficients in Eqs. (S14) and (S15) depend on k_c. A good approximation is obtained fitting separately the parameters for $k_c < k_{c,ac}$ and $k_c > k_{c,ac}$.

The nonzero value of k_c^{min} is due to the ATP mantainence flux, which forces the carbon intake flux to be strictly positive also at zero growth rate. We see that $k_c^{\min} \ll K_M^c$, implying that the the mantainence flux only affects the growth rate at low growth rates. Most importantly, we see that when carbon overflow sets in the cell switches to a metabolism with larger maximum growth rate, $\lambda_{\max} = \mu \times (\phi_Q^{\max} - \phi_Q)$, but smaller affinity for the carbon source (that is, a larger Michaelis constant K_M^c). This is in agreement with the idea that the cell should use low-quality carbon nutrients very efficiently (with the highest possible growth yield), whereas it should grow as fast as possible in presence of high-quality nutrients.

Proteome fractions

The effect of carbon limitation and translational inhibition on the optimal proteome allocation is summarized in Main Figure 1. Results match those obtained in Ref. [14]. In particular, the ribosomal proteome fraction ϕ_R is found to anticorrelate with the growth rate upon antibiotic-induced translational limitation [14]. This is also consistent with Eq. (S13), as one can easily verify upon expressing k_r as a function of λ for fixed k_c.

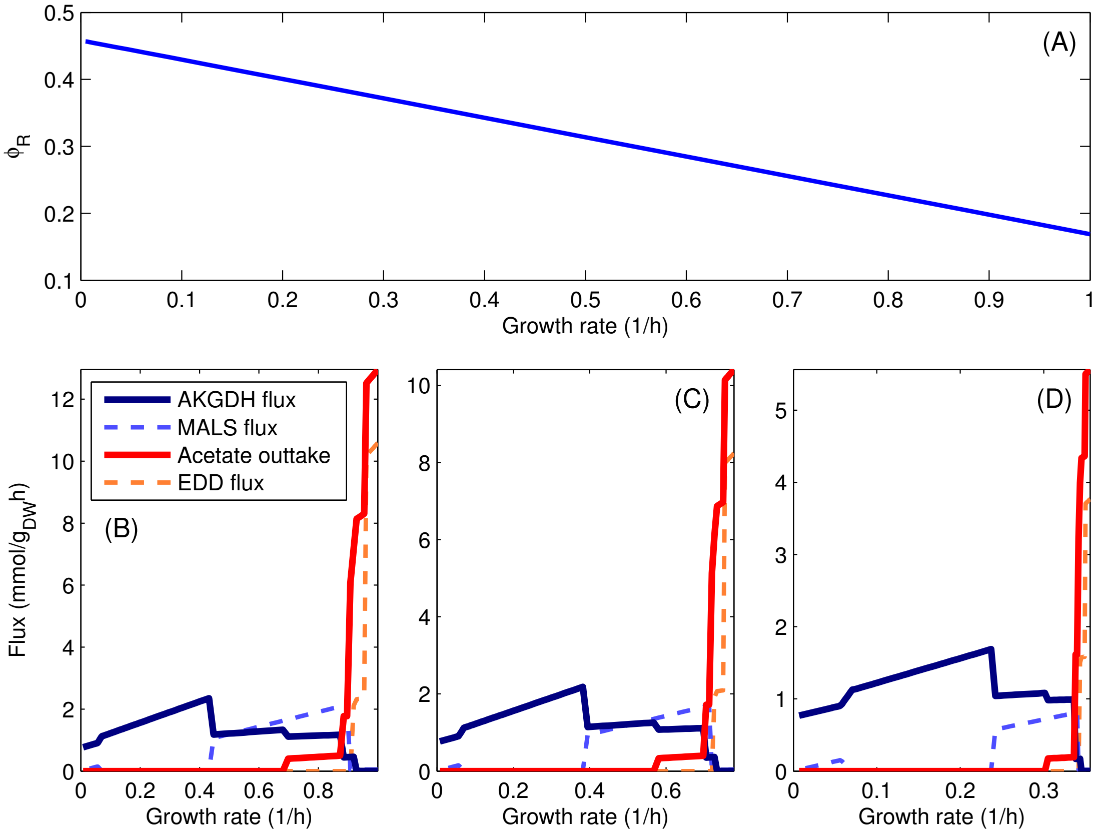

Figure N4: (**A**): ribosomal proteome fraction ϕ_R as a function of growth rate $\lambda_{\max}$, obtained by solving CAFBA with $w_C = 0$ and varying $w_R = 1/k_r$ (translational limitation). Bottom panels: fluxes as a function of λ for different values of w_R (from left to right: w_R =0.169 h (**B**), 0.3 h (**C**) and 1 h (**D**)) obtained by varying the degree of carbon limitation through w_C.

Defining $\hat{\lambda} = \lambda_{max}(k_c - k_c^{\min})/(k_c + K_M^c)$ and $\hat{K}_M^r = K_M^r k_c/(k_c + K_M^c)$ we have:

$$\lambda = \hat{\lambda}\frac{k_r}{k_r + \hat{K}_M^r} \quad \longrightarrow \quad k_r = \hat{K}_M^r \frac{\lambda}{\hat{\lambda} - \lambda} \tag{S16}$$

$$\Rightarrow \quad \phi_R = \left(\phi_{R,0} + \frac{\hat{\lambda}}{\hat{K}_M^r}\right) - \frac{\lambda}{\hat{K}_M^r} \tag{S17}$$

We see that $\hat{\lambda}/\hat{K}_M^r$ is only weakly dependent on k_c, so that the y-intercept of ϕ_R is almost constant, $\phi_R \sim \lambda_{max}/K_M^r \sim (\phi_Q^{\max} - \phi_Q)$, since $\mu \sim K_M^r$ (see Table N1). On the other hand, $\hat{K}_M^r$ increases with the nutritional quality k_c, as also shown in Ref. [14].

Fluxes and Q–limitation

Figures N4 and N5 detail how the pattern of single fluxes obtained in carbon limitation change in response to variations of w_R and $\phi_{\max}$, respectively. In both cases fluxes approximately rescale with λ, without any significant modification in the flux patterns. CAFBA predictions for both translational limitation and overexpression of unnecessary proteins (modeled increasing ϕ_Q) therefore reduce approximatively to a rescaling of both growth rate and the fluxes with the same scaling factor, $v_i \propto (\phi_Q^{\max} - \phi_Q)$, with $\phi_Q^{\max} = 91\%$.

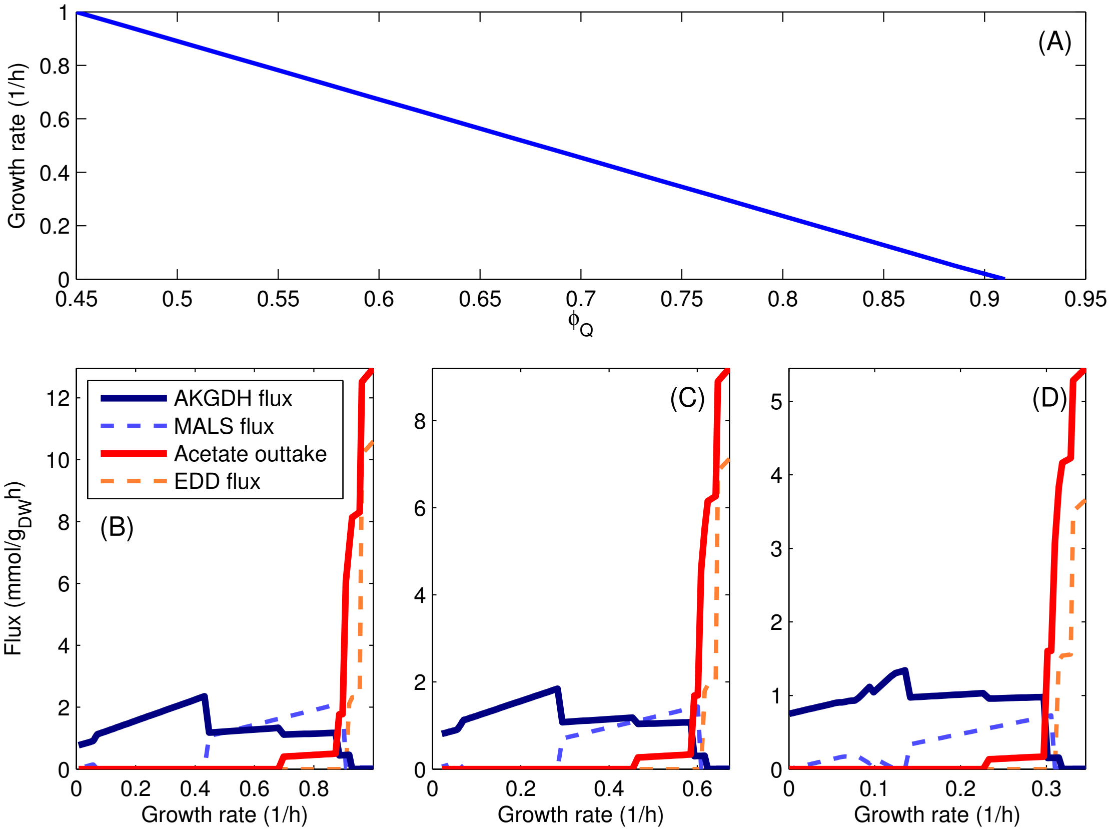

Figure N5: (**A**): growth rate λ in saturating glucose ($w_C = 0$) computed with CAFBA as a function of ϕ_Q. Note that $\lambda = 1.0$/h at $\phi_Q = 0.45$, and decreases linearly with ϕ_Q. **Bottom** panels: fluxes as a function of λ for three different values of ϕ_Q (from left to right: ϕ_Q =45% (**B**), 60% (**C**) and 75% (**D**)) obtained by varying the degree of carbon limitation through w_C.

Note E. Case of inhomogeneous proteome costs

The growth rate–dependent enzymatic proteome fraction reads

$$\Delta\phi_E = \sum_{i \in \text{int.}} w_i |v_i| \quad . \tag{S18}$$

In order to go beyond the homogeneous case $w_i = w_E \equiv 0.00083\ \mathrm{g_{DW} h/mmol}$ for all i, we have considered an inhomogeneous scenario in which w_i's are quenched random variables and final results are obtained by averaging over many instances of CAFBA, each with different choices of w_i's. Here we characterize this scenario more precisely.

We have chosen to draw the logarithm of each weight w_i independently from a box distribution of the form

$$p\big(\log_{10}(w_i)\big) = \begin{cases} \frac{1}{\delta} & \log_{10}(\bar{w}) - \frac{\delta}{2} \le \log_{10}(w_i) \le \log_{10}(\bar{w}) + \frac{\delta}{2} \\ 0 & \text{otherwise} \end{cases} \quad , \tag{S19}$$

where $\bar{w}$ and δ are parameters. This corresponds to a probability density function $p(w_i) \propto 1/w_i$ for $w_i \in [\bar{w} \cdot 10^{-\delta/2}, \bar{w} \cdot 10^{\delta/2}]$. δ representes therefore the number of decades spanned by the weights. The average value $\langle w \rangle$ is related to the parameters $\bar{w}$ and δ by

$$\langle w \rangle = \bar{w} \frac{10^{\delta/2} - 10^{-\delta/2}}{\delta \log_e 10} \quad . \tag{S20}$$

One can see that $\lim_{\delta \to 0} \langle w \rangle = \bar{w}$. We checked for possible biases in our analysis by comparing the results with the ones obtained using a lognormal distribution. As shown in Fig. S3, the two distributions yield similar results, provided that the variance of the logarithms of the weights is the same.

Averages are computed by defining a number of replicas of the model, each one with a different realization of the coefficients $\{w_i\}$, computing the CAFBA solutions for each realization, and then averaging the resulting fluxes. This procedure will also generate a distribution for the growth rate. For small δ, it turns out to be peaked around the growth rate corresponding to $\langle w \rangle = \bar{w}$, but in general the mean growth rate will depend on δ. In practice, the parameter $\bar{w}$ is be chosen as to keep $\langle \lambda \rangle$ to a prescribed value, e.g. 1/h.

Sample to sample fluctuations and fluctuations in the weights

In this section we describe the effect of increasing the width of the fluctuations on the weights w_i. As we noted before, if we only fix the average weight $\langle w \rangle$, the average growth rate depends on δ, making CAFBA solutions more difficult to compare. Therefore, for sakes of clarity, *only in this section* we rescaled the weights w_i such that the maximum growth rate is $\lambda_{\max} = 1$/h for all the different synthetic strains. Fig. N6 shows the sample–to–sample fluctuations in αKG dehydrogenase flux and in acetate excretion, along with the average values. The top panels (**A**) and (**B**) show the samples obtaines without rescaling the strains, while the bottom panels (**C**) and (**D**) are produced using such procedure. The results are very similar, although the rescaling reduces dramatically the variance of the growth rate at fixed w_C.

We have furthermore carried out a systematic study of how CAFBA solutions depend on the parameter δ. In particular, we have studied the averaged CAFBA solutions in glucose minimal medium, fixing the maximum growth rate to 1/h and using different values of δ, ranging from 0 (no randomization) to 4 ($w_{\max}/w_{\min} = 10^4$). Results are shown in Fig. N7 and N8. As the width δ of fluctuations is increased, transitions between pathways are progressively smoothed. For $\delta \sim 1$ the average acetate excretion grows as a linear function of growth rate, starting from $\lambda \sim 0.7$/h. As δ is increased above 1, a general decrease in growth yield is observed, with increasing average glucose uptake and excretions of intermediate metabolites (mainly dihydroxyacetone and formate). Moreover, solutions are highly heterogeneous from sample to sample, as measured by the standard deviation of the fluxes σ_v. This quantity is obtained by first computing the standard deviation of sample–to–sample fluctuations of a single flux, and then averaging over all reaction whose weight w_r is larger than zero:

$$\sigma_v = \frac{1}{N_r} \sum_{r: w_r > 0} \sigma_v^r \quad \text{with} \quad \sigma_v^r = \sqrt{\left(\frac{1}{N_s}\sum_s v_{r,s}^2\right) - \left(\frac{1}{N_s}\sum_s v_{r,s}\right)^2} \tag{S21}$$

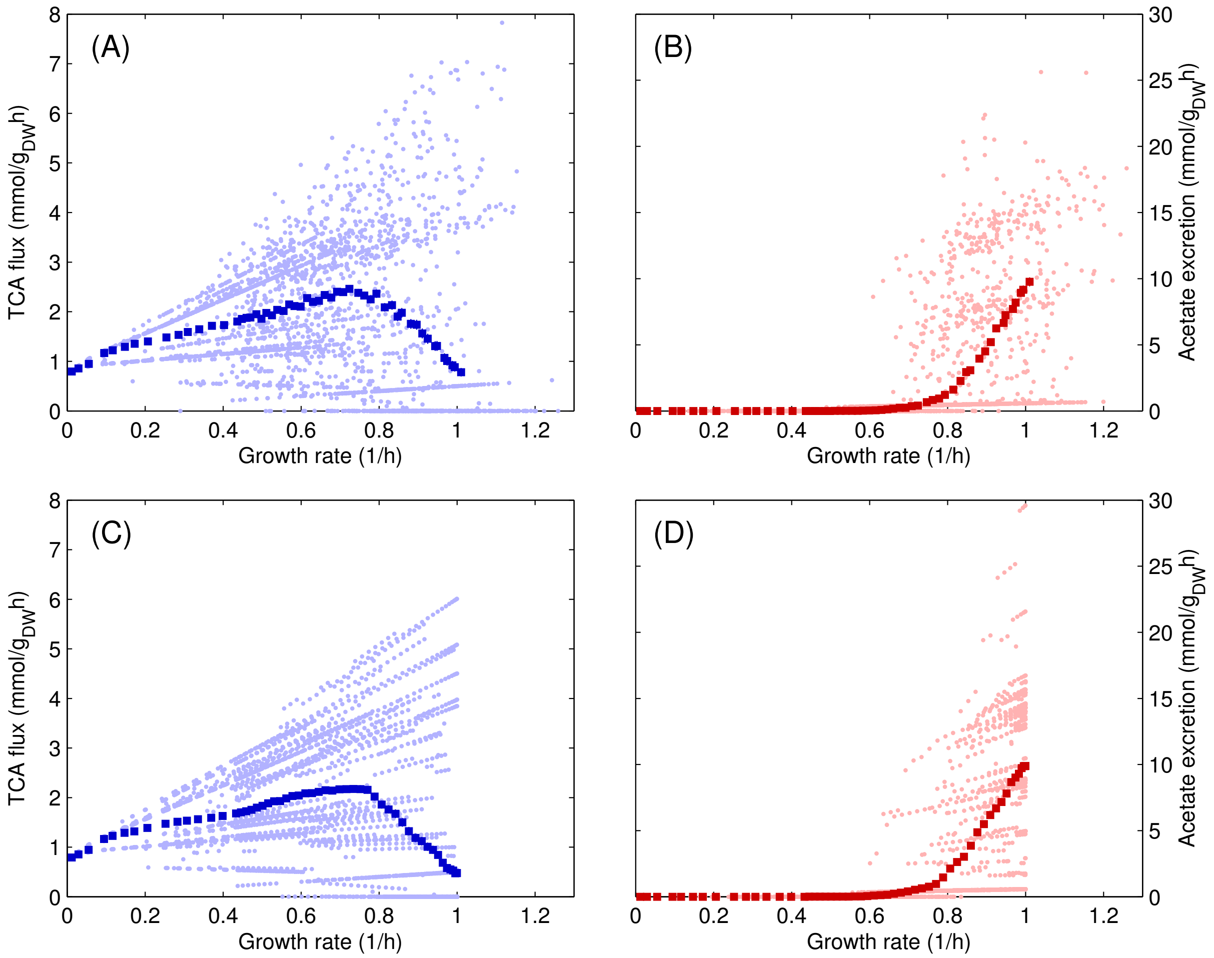

Figure N6: Sampled fluxes for the randomized models, using $\delta = 1$. The pale dots are the actual sampled fluxes, while the squares are the average of fluxes for a given w_C. Each of the $N_s = 500$ copies of the model has been sampled for 54 different values of w_C. Top panels (**A** and **B**): Indipendent extractions ($N_p \times N_s$) of the weights. Bottom panels (**C** and **D**): Each of the 500 copy of the model had the weights rescaled such that maximum growth rate is 1/h. Both procedures yield similar results, apart for the fluctuations in the growth rate. Only a subset of the sampled fluxes (50 out of 500 for each value of w_C) is shown for clarity. See text for details.

where v_r^s is the flux of reaction r in the sample s. As shown in Fig. N8, the heterogeneity of the fluxes increases with δ and with growth rate (since fluxes are generally proportional to growth rate itself).

In conclusion, we observe that exmpirical evidence is better reproduced by values of δ close to one, and we will therefore use this value throughout the rest of the study.

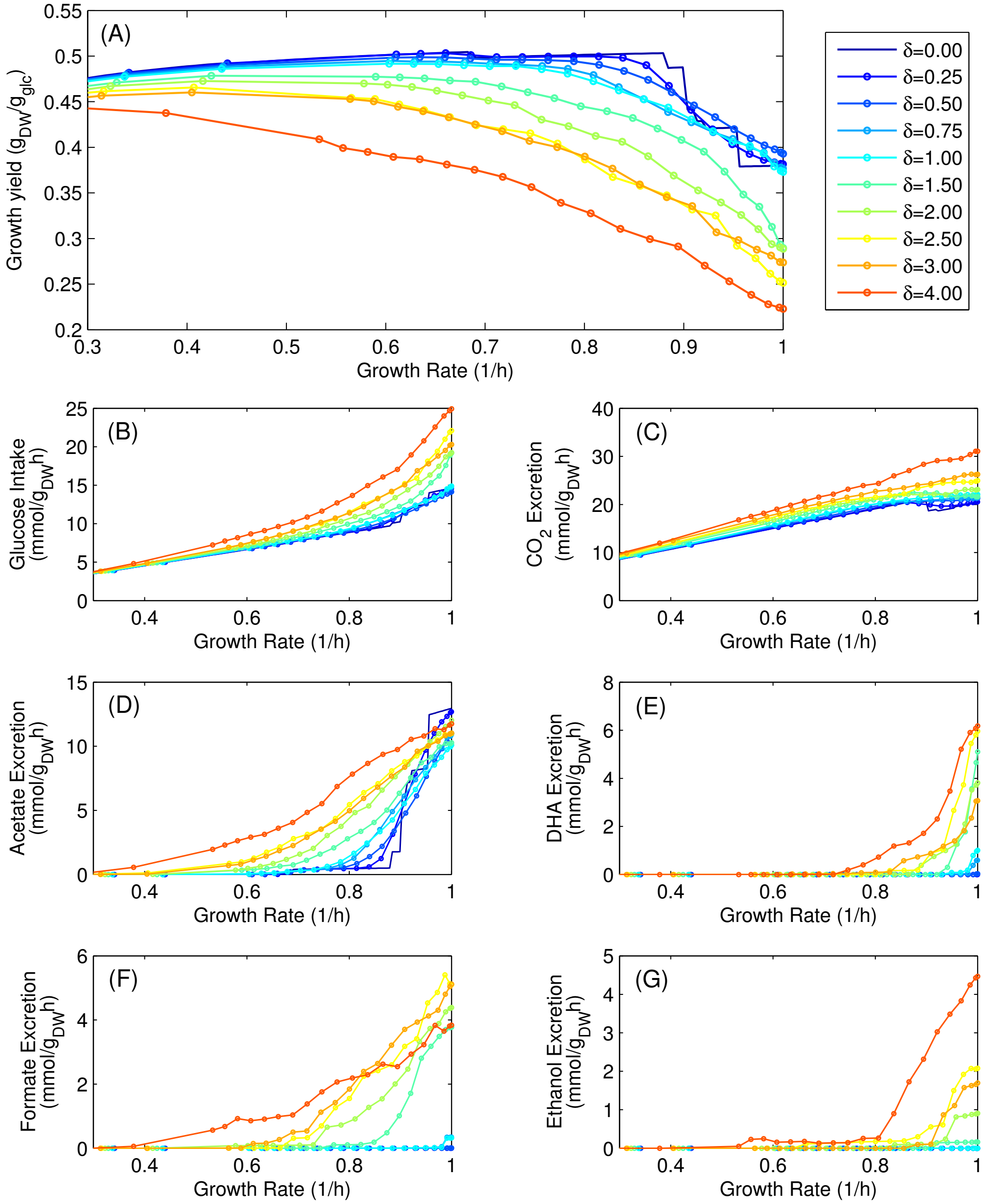

Figure N7: Average value of growth yield and exchange fluxes versus growth rate, for different values of $\delta = \log_{10}(w_{\max}/w_{\min})$, from top to bottom: (**A**) growth yield, (**B**) glucose uptake, (**C**) CO2 excretion, (**D**) acetate excretion, (**E**) formate excretion, (**F**) dihydroxiacetone (DHA) excretion and (**G**) ethanol excretion. The $\delta = 0$ case corresponds to the non-randomized case. For each value of w_C and δ, the averages fluxes (using 200 samples for each point) are indicated with circles (error bars not shown for clarity). Weights have been reascaled as to fix the maximum growth rate to 1/h.

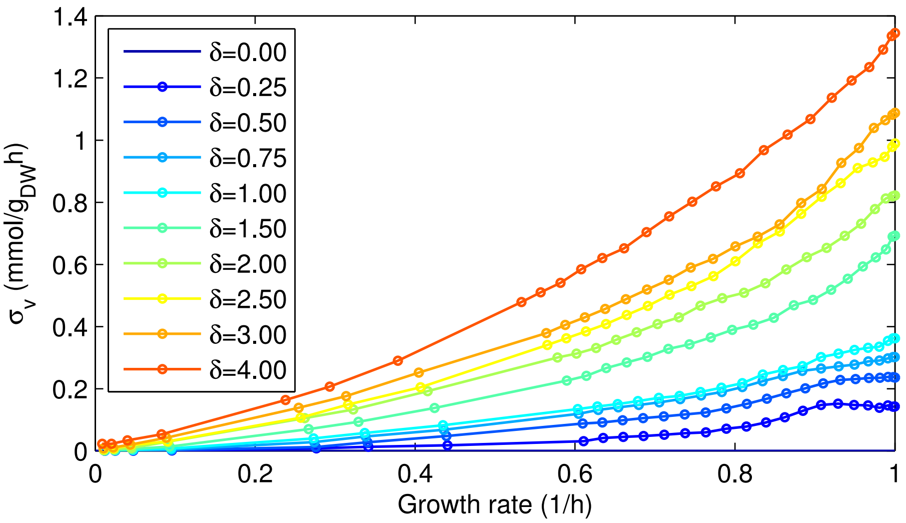

Figure N8: Average standard deviation σ_v of the fluxes, as a function of the average growth rate for different values of δ. Flux variability increases with both growth rate and δ.

Note F. Growth-dependent biomass composition

Growth rate–dependent biomass composition produces a nonlinear optimization problem, which is no longer solvable using the standard linear programming tools. We will first show how one can account for a growth–rate dependent biomass composition, and then we will describe how it can be straightforwardly implemented as a sequence of LP problems in Sect. .

A growth–rate dependent biomass composition can be easily accounted for in CAFBA and, more in general, in constraint–based metabolic models. Let us denote by β_μ the stoichiometric coefficient corresponding to the amount of metabolite μ required to build one gram of dry weight (corresponding, for instance, to the coefficients given in the iJR904 model). The mass fraction of metabolite μ, ψ_μ, is obtained multiplying β_μ by μ's molecular mass. Because the cell's composition changes with λ, β_μ's are, in general, functions of the growth rate. For sakes of simplicity, we have characterized the biomass composition by dividing biomass components and their corresponding β_μ in four classes:

- β_{AA} is the total amount of amino acids per unit of dry weight;
- β_{DNA} is the total amount of DNA nucleotides (DATP, DCTP, DGTP,DTTP) per unit of dry weight;
- β_{RNA} is the total amount of RNA nucleotides (ATP,CTP,GTP,UTP) per unit of dry weight;
- β_{LIP} denotes the amount of lipids, liposaccharides and similar biomass constituents (see Table N2).

The individual stoichiometric coefficients for metabolite μ change with λ as

$$\beta_\mu(\lambda) = \beta_{X[\mu]}(\lambda)\frac{\beta_\mu}{\sum_{\nu\in X[\mu]}\beta_\nu} \ , \qquad \text{(S22)}$$

where $X[\mu] \in \{AA, RNA, DNA, LIP\}$ denotes the class to which metabolite μ belongs.

All remaining stoichiometric coefficients for biomass components are chosen as in the iJR904 model. They are mainly cofactors or other components with very small biomass fractions: 5–methyltetrahydrofolate, acetyl–CoA, CoA, FAD, NAD, NADH, NADP, NADPH, Succinyl–CoA and UDP–glucose. Together, they account for a mass M_{other} which is roughly $\sim 2.5\%$ of total dry weight mass.

In order to provide plausible β functions we have to consider two constraints. Let us define $\psi_X = M_X/M_{DW}$. These quantities are related to the β functions through the molecular masses of the biomass

components. The first one is the normalization of the total mass of the cell:

$$\psi_{AA} + \psi_{DNA} + \psi_{RNA} + \psi_{LIP} = 1 - \psi_{other} \ . \tag{S23}$$

The second constraint is the linear relation between the RNA/protein mass ratio and growth rate λ [18]. The biomass functions $\beta(\lambda)$ we choose must satisfy such empirical constraint, which can be written as [14]:

$$R(\lambda) = \psi_{RNA}/\psi_{AA} = r_0 + \lambda/\kappa_t \ , \qquad r_0 = 0.087 \pm 0.009 \ , \qquad \kappa_t = 4.5 \pm 0.2/\mathrm{h} \ . \tag{S24}$$

Therefore, we have to fix two ψ functions, for instance ψ_{DNA} and ψ_{LIP}, while the other two can be computed using using the constraints Eqs. (S23) and (S24). These four ψ–functions are listed in Table N3 and plotted against growth rate in Fig. N11.

Implementation of a growth-dependent biomass composition in CAFBA

Growth rate–dependent biomass coefficients cannot be directly included in CAFBA (or FBA), since the resulting problem is no longer linear. On the other hand, one can treat this case as a sequence of LP problems with constant biomass composition along the following lines:

1. Initiate by fixing a growth rate λ_0 and computing the biomass coefficients $\beta(\lambda_0)$, e.g. via the prescriptions shown in Table N3 for the main biomass groups and Eq. (S22) for the individual stoichiometric coefficients.

2. At each step k, compute the growth rate λ_k by solving CAFBA with biomass coefficients $\beta(\lambda_{k-1})$;

3. Iterated until $\lambda_k - \lambda_{k-1}$ is smaller than a fixed threshold (in our case, 10^{-4}/h).

This method is found to converge very rapidly to the optimal solution, see Fig. S9. In particular, the difference $|\lambda_k - \lambda_{k-1}|$ decreases exponentially with the number of steps k. This procedure is performed keeping all other parameters fixed. For instance, in the case of carbon limitation, the procedure must be performed individually for each value of w_C. A good initial guess λ_0 for the growth rate can speed up the calculation, although providing an intermediate value (e.g. 0.6/h for all w_C's) works well in practice.

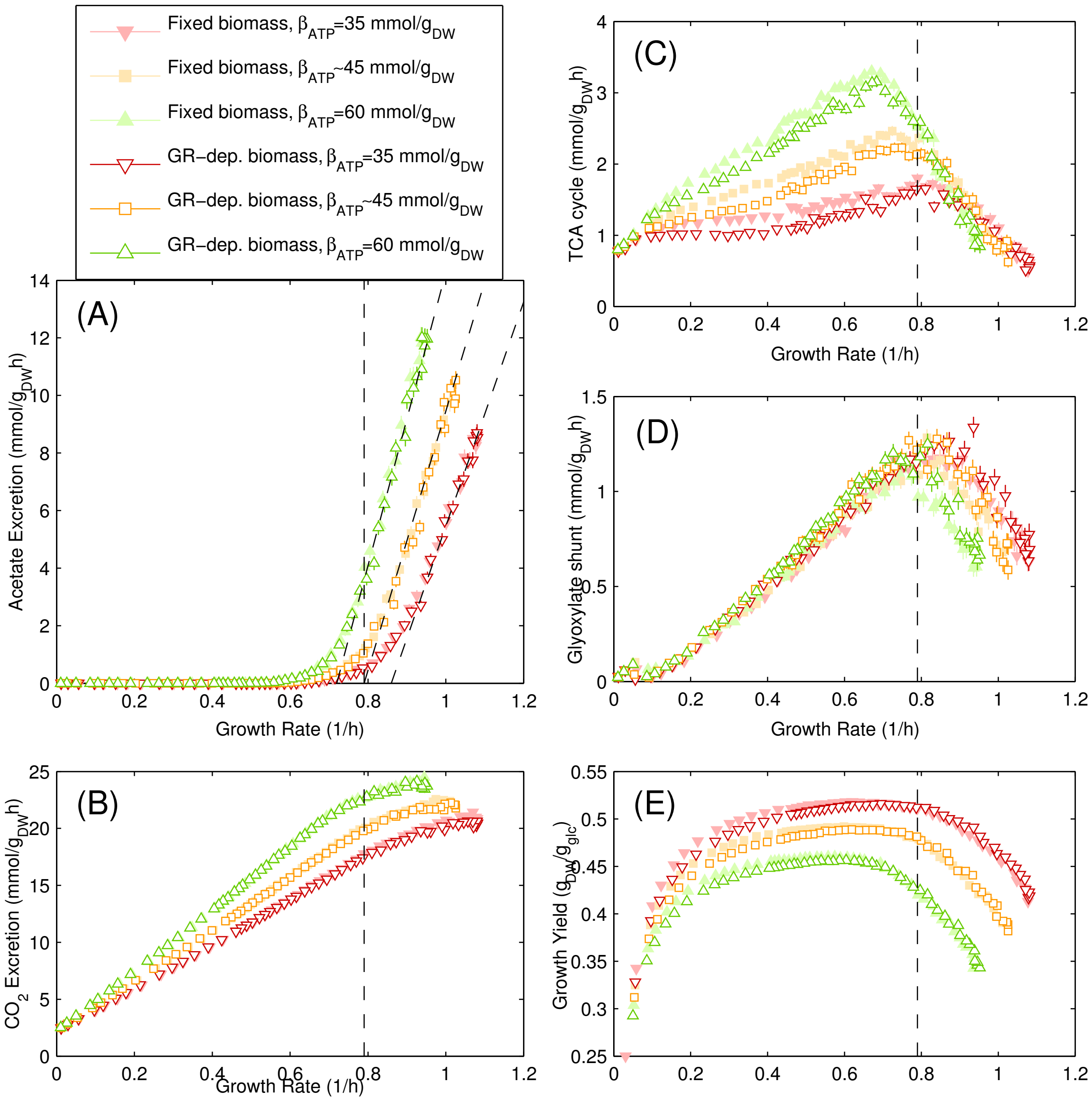

Figure N9: Representative fluxes obtained by CAFBA for *E. coli* growth in glucose minimal medium with constant (filled red, yellow and green markers) and variable (in open red, yellow and green markers) biomass composition for three different values of the λ-dependent ATP hydrolysis rate $\beta_{\rm ATP}$. (A) Acetate secretion rate, (B) CO_2 secretion rate, (C) flux through TCA cycle (αKG dehydrogenase), (D) flux through glyoxylate shunt (Malate synthase), (E) growth yield. No significant differences are observed between the constant and λ-dependent cases for $\beta_{\rm ATP} = 45.5608\ {\rm mmol_{ATP}/g_{DW}}$, corresponding to the default value for the iJR904 model. We also show, for comparison, results obtained for larger and smaller values of $\beta_{\rm ATP}$. The acetate secretion rate can always be fitted by a linear function of λ, i.e. $v_{\rm ac} = s \times (\lambda - \lambda_{\rm ac})$, albeit with different slopes and intercepts. The three dashed lines correspond to $s = 39, 45, 51\ {\rm mmol/g_{DW}}$, respectively, while $\lambda_{\rm ac} = 0.86, 0.79, 0.72$/h, respectively. We also indicate $\lambda_{\rm ac} = 0.79$/h with a vertical dashed line in all panels. In all cases we set $\langle w \rangle = 8.8 \times 10^{-4}$ gh/mmol, $w_C \geq 0$ and $w_{\rm max}/w_{\rm min} = 10$.

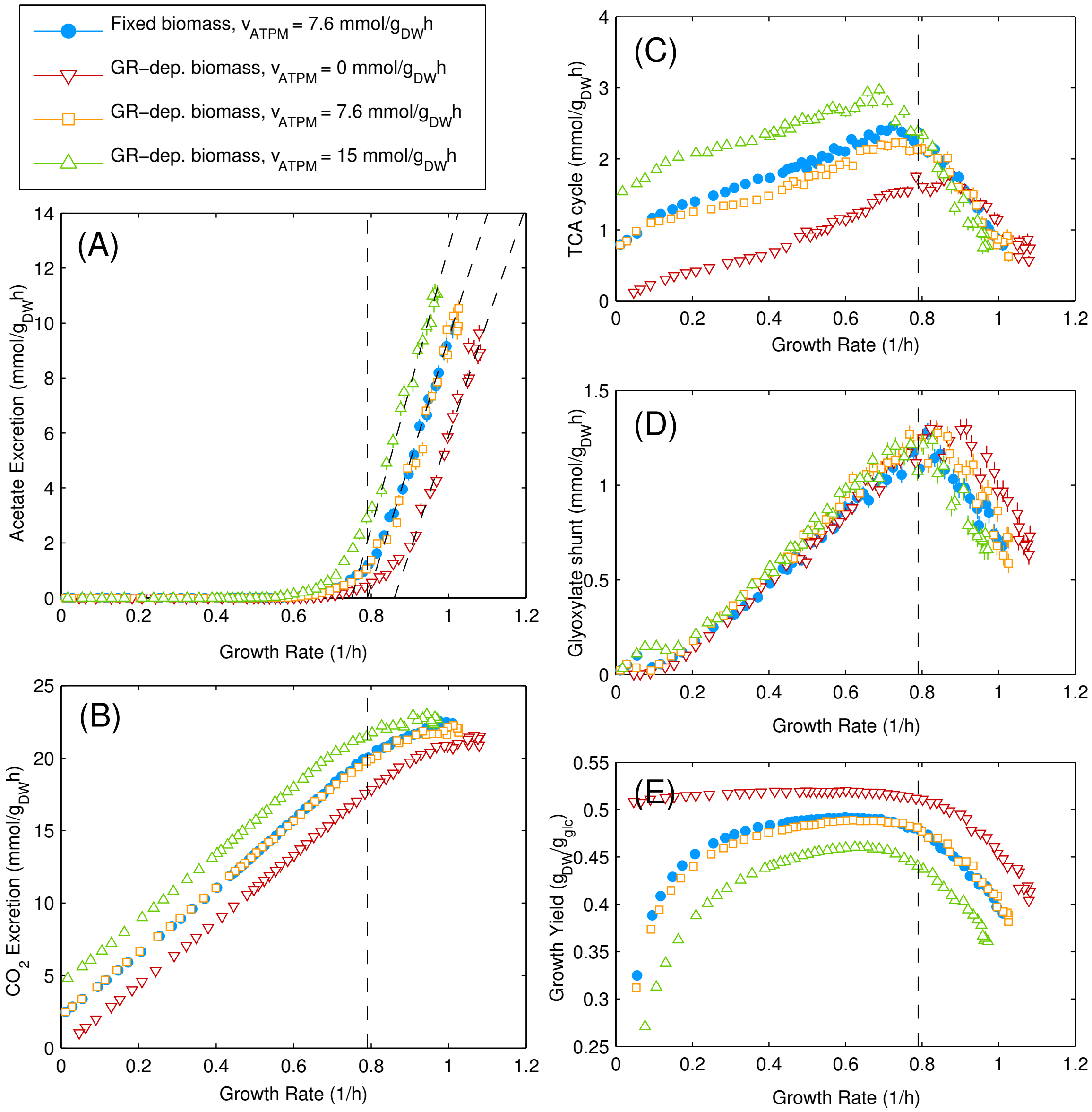

Figure N10: Representative fluxes obtained by CAFBA for *E. coli* growth in glucose minimal medium with constant (filled red, yellow and green markers) and variable (in open red, yellow and green markers) biomass composition for three different values of the λ-independent ATP hydrolysis rate v_{ATPM}). (A) Acetate secretion rate, (B) CO_2 secretion rate, (C) flux through TCA cycle (αKG dehydrogenase), (D) flux through glyoxylate shunt (Malate synthase), (E) growth yield. Results are similar to the case shown in Fig. N9, where β_E is varied instead. The acetate secretion rate can always be fitted by a linear function of λ, i.e. $v_{\mathrm{ac}} = s \times (\lambda - \lambda_{\mathrm{ac}})$, albeit with different slopes and intercepts. The three dashed lines correspond to $s = 42, 45, 51$ mmol/g_{DW}, respectively, while $\lambda_{\mathrm{ac}} = 0.86, 0.79, 0.75$/h, respectively. We also indicate $\lambda_{\mathrm{ac}} = 0.79$/h with a vertical dashed line in all panels. In all cases we set $\langle w \rangle = 8.8 \times 10^{-4}$ gh/mmol, $w_C \geq 0$ and $w_{\max}/w_{\min} = 10$.

#	Name	β (mmol/g$_{\mathrm{DW}}$)	m (g/mol)	Mass fraction (%)
227	Cardiolipin (LP)	0.000129	69708	0.9
576	Phosphatidylethanolamine (PE)	0.001935	35656	6.9
580	Phosphatidylglycerol (PG)	0.000464	37155	1.72
614	Phosphatidylserine (PS)	0.000052	37805	0.20
406	Glycogen	0.154	162	2.5
489	Lipopolisaccharide	0.0084	3877	3.25
579	Peptidoglycan subunit (murein)	0.0276	990.4	2.73
616	Putrescine	0.035	90	0.315
662	Spermidine	0.007	148	0.1

Table N2: Stoichiometric coefficients β of lipids (LP+PE+PG+PS), lipopolysaccarides, glycogen, murein, putrescine and spermidine in the iJR904 model (the first column gives the metabolite's ID in the reconstruction).

Function (mmol/g$_{\mathrm{DW}}$)	Reference
$\psi_{DNA}(\lambda) = 0.06\dfrac{1+\lambda^2}{1+3\lambda^2}$	See Ref. [19]
$\psi_{LIP}(\lambda) = \dfrac{0.27+0.14\lambda}{1+2\lambda}$	See Ref. [20]
$\psi_{AA}(\lambda) = \dfrac{1}{1+R(\lambda)} \times [1-\psi_{other}-\psi_{DNA}(\lambda)-\psi_{LIP}(\lambda)]$	See Refs. [20] (low growth rates) and [21] (high growth rates)
$\psi_{RNA}(\lambda) = \dfrac{R(\lambda)}{1+R(\lambda)} \times [1-\psi_{other}-\psi_{DNA}(\lambda)-\psi_{LIP}(\lambda)]$	See Refs. [20] (low growth rates) and [21] (high growth rates)

Table N3: Formulas for the mass fractions ψ of various biomass groups: proteins (AA), RNA, DNA and lipids (LIP). Here $R(\lambda)$ is the experimental RNA/protein mass ratio, Eq. (S24). In all formulas, λ is to be expressed in units of 1/h. The functions are plotted in Fig. N11. In the "Reference" column some references which were used to build the functions are shown. We also use $\psi_{other} = 2.5\%$.

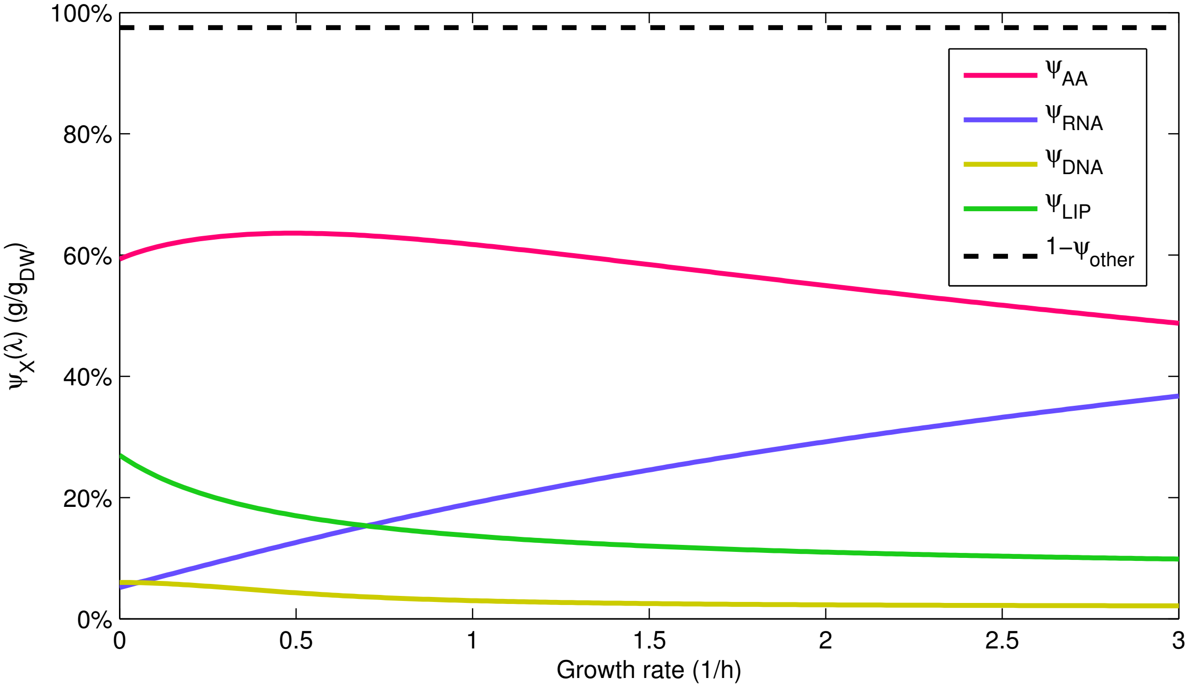

Figure N11: Plots of the four $\psi(\lambda)$ functions (ψ_{AA}, ψ_{RNA}, ψ_{DNA}, ψ_{LIP}) which fix the mass fraction of each biomass sector. The functions are further described in the text and in Table N3.

Supplementary Tables

Captions are in the main text file.

Carbon source	$\lambda_{\max}$ (1/h)
Glucose	1.000
Lactose	0.984
Mannose	0.987
Maltose	0.991
Sucrose	1.005
Galactose	0.990
Fructose	1.000
Mannitol	1.009
Sorbitol	1.010
Glucose-6P	1.075
Mannose-6P	1.075

Table A: Extrapolated maximum growth rates $\lambda_{\max}$ for a variety of carbon sources using $w_C = 0$ and $w_E = 8.3 \times 10^{-4}$ $\mathrm{g_{DW}h/mmol}$. In this conditions the proteome fraction of the C-sector is at its minimum, $\phi_C = \phi_{C,0}$ and $\Delta\phi_C = 0$, while growth is limited by the E– and R–sectors only. In this case the CAFBA constraint reads $\Delta\phi_E + \Delta\phi_R = \phi_{\max}$ with $\Delta\phi_E = \sum_r w_r|v_r|$ and $\Delta\phi_R = w_R\lambda$. $\lambda_{\max}$ is, by definition, the x–axis intercept of the C–line (see Fig. 1B). All non–phosphorylated carbon sources have similar C–lines, as seen by $\lambda_{\max}$ consistently being between 0.984 and 1.01/h. Glucose–6P and mannose–6P can provide a larger maximum growth rate due to the extra ATP they generate per flux unit. CAFBA solutions are computed using $w_i = w_E = 8.3 \times 10^{-4}$ $\mathrm{g_{DW}h/mmol}$ for all E–sector reactions, $w_R = 0.169$ h and $\phi_{\max} = 48.4\%$.

Carbon source	$\lambda_{\exp}$ (1/h)	$w_{C,0}$ ($\mathrm{g_{DW}h/mmol}$)	$\Delta\phi_C$
Mannose	0.41	5.67×10^{-2}	26.4%
Sorbitol	0.46	5.09×10^{-2}	24.6%
Fructose	0.61	2.57×10^{-2}	17.3%
Maltose	0.67	3.8×10^{-2}	14.0%
Glucose	0.85	6.38×10^{-3}	6.0%
Lactose	0.98	2.7×10^{-4}	0.2%

Table B: Carbon limitation is modeled in CAFBA by varying the parameter w_C, which sets the C–sector fraction per carbon flux unit, or C–sector weight. We report here a set of experimental growth rates $\lambda_{\exp}$ from Ref. [16], obtained for an *E. coli* NCM3722 strain in batch culture for different carbon sources (minimal medium). For each carbon source a lower bound $w_{C,0}$ to the C-sector weight exists, so that CAFBA growth rate equals $\lambda_{\exp}$ at $w_C = w_{C,0}$. Reduced external concentrations of the nutrient are modeled by using an increased value of $w_C \geq w_{C,0}$, as described in the Main Text. In the last column we show the value of $\Delta\phi_C = w_C v_C$ at $w_C = w_{C,0}$, i.e. at the maximum growth rate allowed for the specific carbon source.

Model	Growth indep. ATP hydr. flux (v_{ATPM})	Growth dep. ATP hydrolysis (β_{ATP})
iJR904	7.6 $\mathrm{mmol/g_{DW}h}$	45.5608 $\mathrm{mmol_{ATP}/g_{DW}}$
iAF1260	8.39 $\mathrm{mmol/g_{DW}h}$	59.806 $\mathrm{mmol_{ATP}/g_{DW}}$
iJO1366	3.15 $\mathrm{mmol/g_{DW}h}$	53.95 $\mathrm{mmol_{ATP}/g_{DW}}$

Table C: *E. coli* metabolic models include an ATP hydrolysis flux $v_{ATP} = v_{ATPM} + \beta_{ATP}\lambda$ to model the energy requirements of the cell. We show here the values of v_{ATPM} and of β_{ATP} for the three models $iJR904$ [22], $iAF1260$ [23] and $iJO1366$ [24].

Supplementary Figures

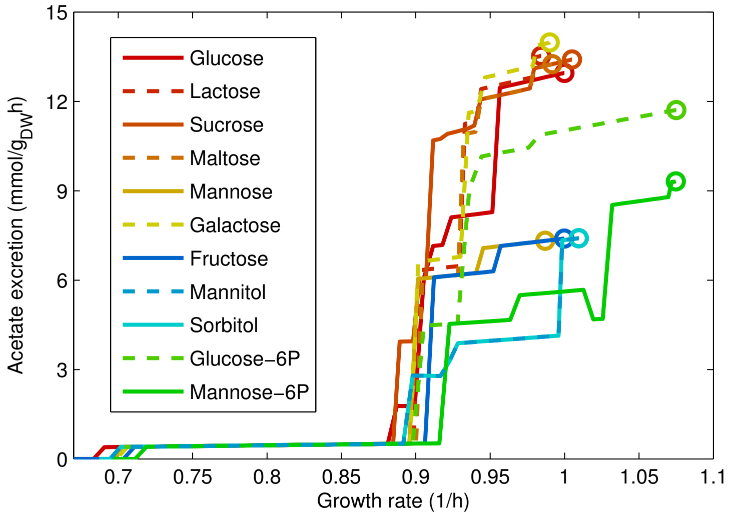

Figure A: Acetate excretion for a variety of carbon sources with uniform weights plotted against growth rate, using $w_E = 8.3 \times 10^{-4}$ $\text{g}_{\text{DW}}\text{h/mmol}$ and $w_C \geq 0$. In particular, the circles indicate the acetate production at the maximum growth rate $\lambda_{\max}$, obtained for $w_C = 0$. See Table A for the numeric values of $\lambda_{\max}$.

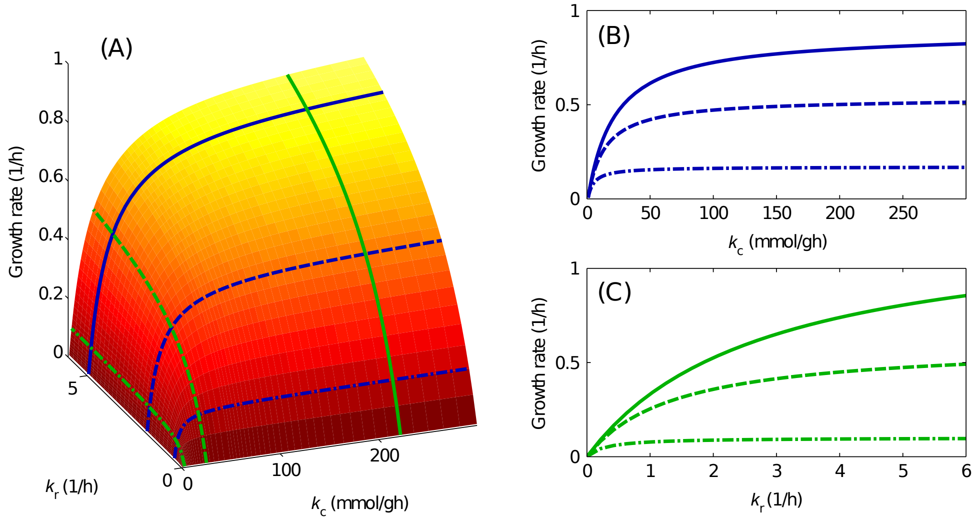

Figure B: (A) Growth rate of CAFBA solutions (minimal glucose medium, homogeneous case using $w_E = 8.3 \times 10^{-4}$ $g_{DW}h/mmol$) as a function of the two parameters $k_c = 1/w_C$ (*nutritional quality*) and $k_r = 1/w_R$ (*translational capacity*). Growth rate is a continuous function of both k_c and k_r, while acetate excretion presents discontinous transitions at different k_c values. Growth rate can be approximated as a Michaelis–Menten function of k_c and k_r, as described in detail in Supplementary note S4. (B) Growth rate plotted as a function of k_c for three fixed values of k_r. These lines correspond to growth rates obtained in C–limitation for different, fixed, amounts of translation limiting antibiotics. The lines are also shown in panel (A) with the same colors and line style. (C) Same as panel (B), but plotting growth rate as a function of k_r for fixed k_c. In this case the lines represent the growth rates obtained in R-limitaiton for different carbon nutrient qualities.

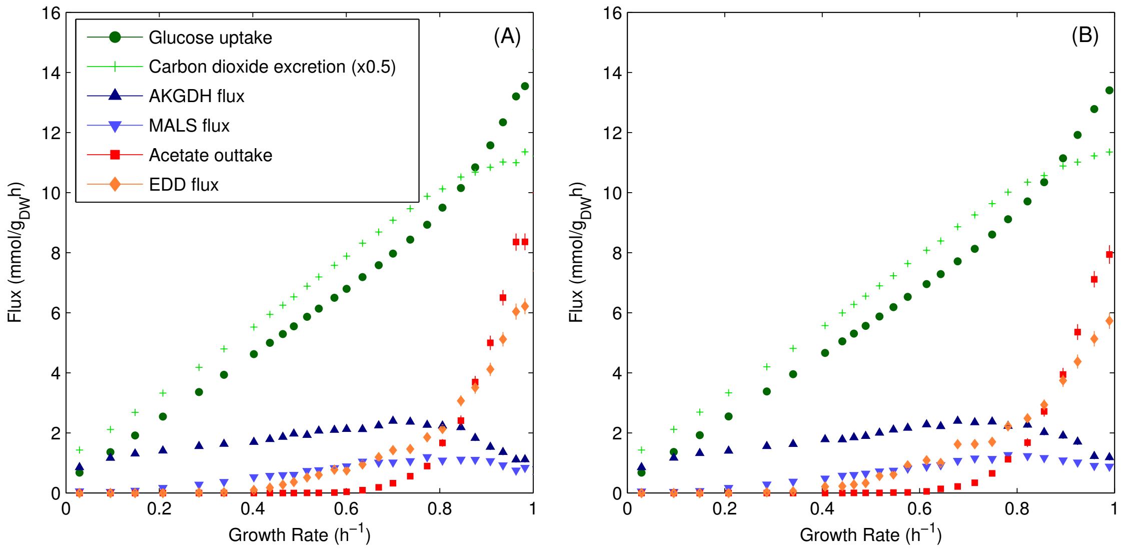

Figure C: Here we compare two sets of CAFBA solutions (500 samples per each value of w_C) computed using different distributions for the E–sector weights w_i. Panel (A): $\log_{10} w_i$ is extracted uniformly in an interval $\log_{10} w_{\min} \leq \log_{10} w \leq \log_{10} w_{\max}$, with $\delta = \log_{10} w_{\max} - \log_{10} w_{\min} = 1$, independently for each i. Note that the variance of this distribution is $\delta^2/12 = 1/12$. Panel (B): the E–sector weights w_i are independently extracted from a lognormal distribution with variance $1/12$, the same as the previous case. In both cases, the average values of the distribution have been tuned such that $\langle \lambda \rangle = 1/h$ when $w_C = 0$. The two distributions yield equivalent results.

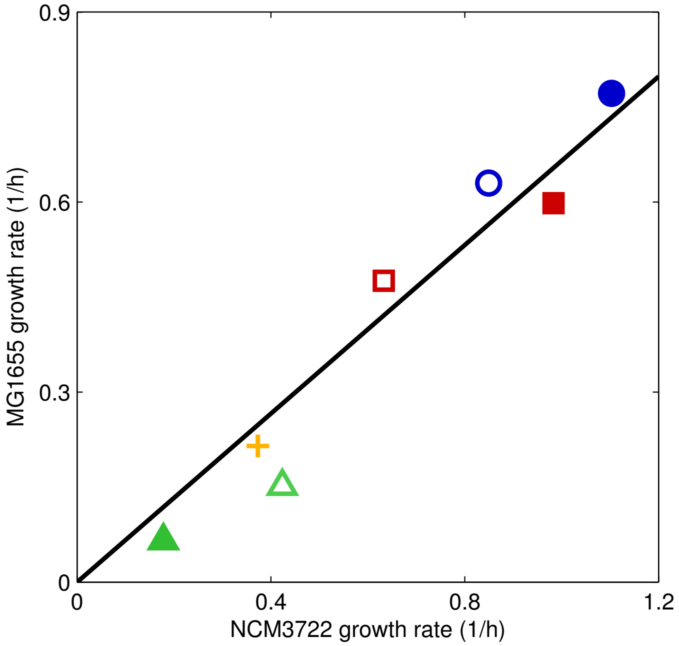

Figure D: Growth rates for *E. coli* K-12 strains NCM3722 and MG1655 in batch cultures at 37°C with vigorous shaking. $N^{-}C^{-}$ minimal medum (see Ref. [16]) was supplemented with the following carbon and nitrogen sources (all in saturating amounts): acetate and NH_4Cl (+), lactose and NH_4Cl (■), glycerol and NH_4Cl (□), glucose-6P, gluconate and NH_4Cl (•), glucose and NH_4Cl (∘), glucose and arginine (▲), glucose and aspartate (△). The best-fit line $\lambda_{MG} = s\lambda_{NCM}$ is shown in the figure, where $s = 0.6652 \sim 2/3$ is the best fit value for the slope.

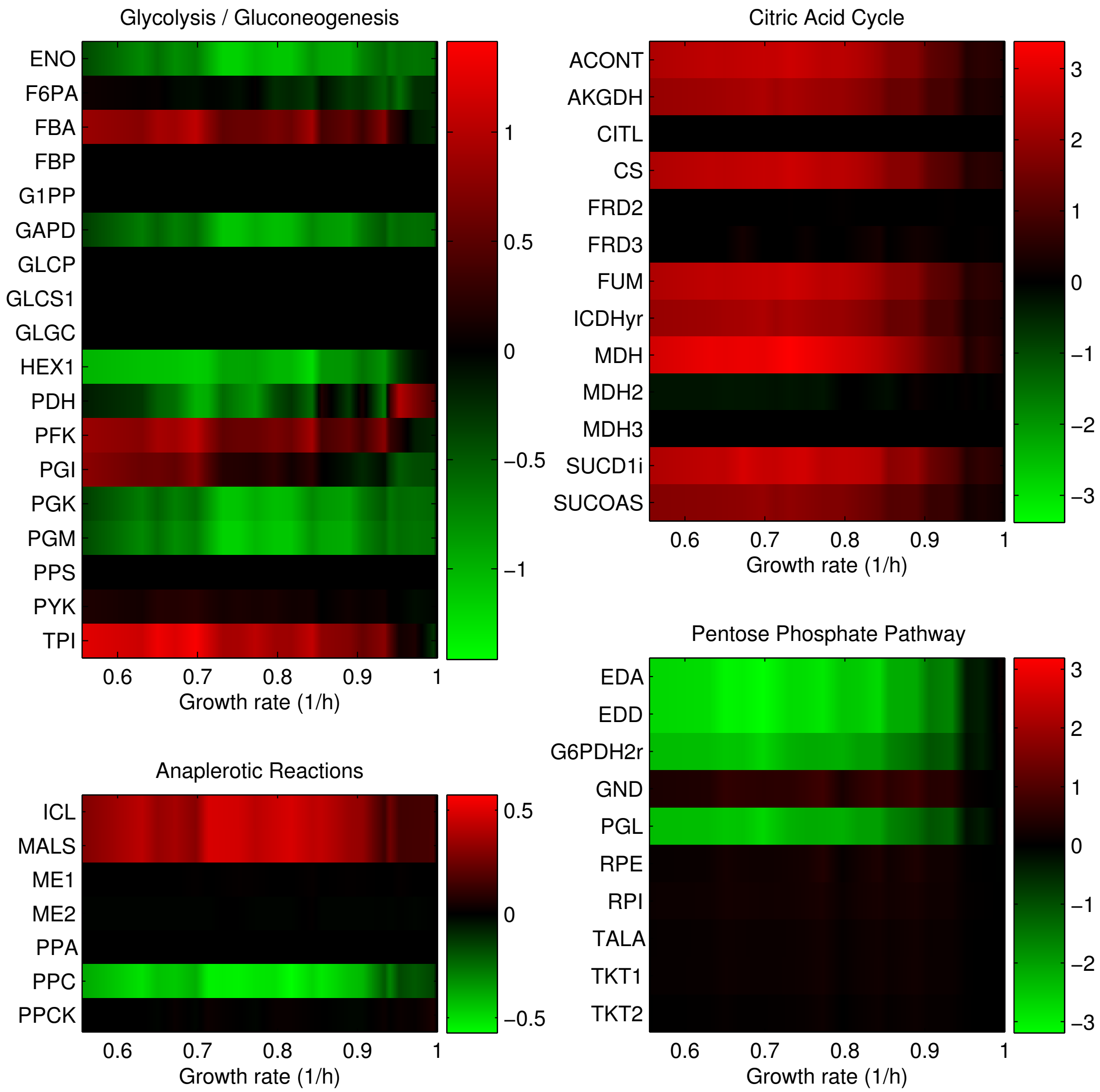

Figure E: Flux differences Δv (in mmol/$g_{\rm DW}$h units) for the main catabolic pathways in glucose minimal substrate. The flux differences are computed from the average fluxes $\langle v_i \rangle$ and growth rates $\langle \lambda \rangle$ (200 samples for each value of w_C) as $\Delta v_i = \langle v_i \rangle - v_i^{\rm ref} = \langle v_i \rangle - \langle v_i \rangle(\lambda_{\rm max}) \cdot (\lambda/\lambda_{\rm max})$, so that Δv_i is zero at the maximum growth rate $\lambda_{\rm max} = 1/h$. Δv_i is larger than zero (shades of red) if the flux $\langle v_i \rangle$ is larger than the reference flux $v_i^{\rm ref} \propto \lambda$ upon carbon limitation, suggesting an upregulation of the corresponding enzymes.

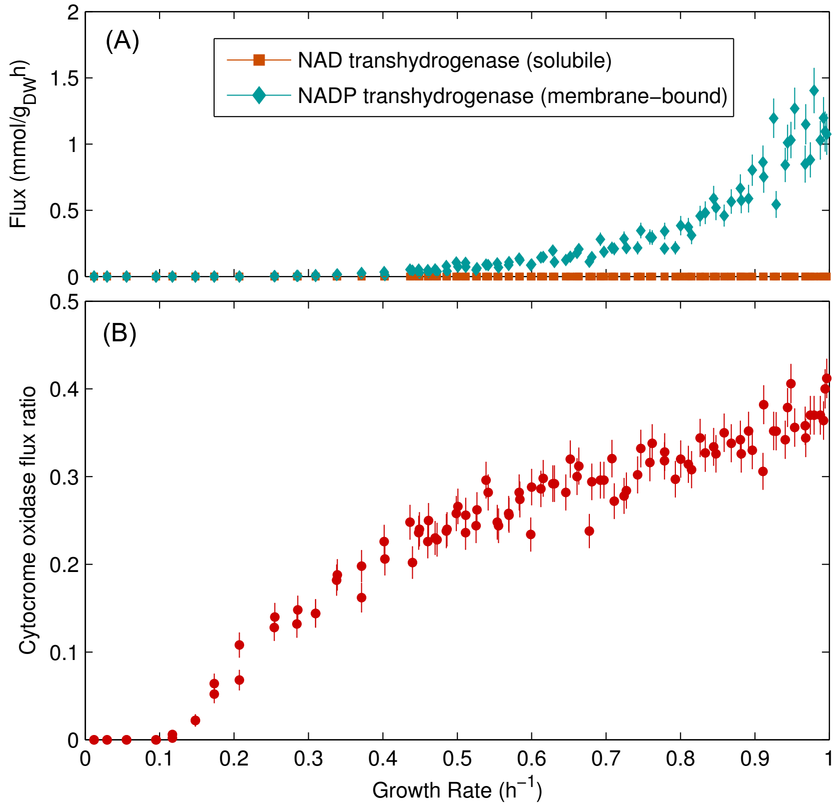

Figure F: (A): The *iJR*904 *E. coli* model contains two distinct NAD(P)H transhydrogenase reactions, whose labels in the model are NADTRHD (NAD transhydrogenase) and THD2 (energy-dependent, membrane-bound, NADP transhydrogenase). The two enzymes are found to be differently regulated, depending on the redox state of the cell [25]. CAFBA solutions show an activation of the THD2 flux at high growth rates, in agreement with experiments. (B): The *iJR*904 *E. coli* model contains two distinct ubiquinol oxidase reactions, CYTBD (*bd*–I enzyme) and CYTBO3 (bo_3 enzyme). The two reactions differ in the proton stoichiometry, as CYTBO3 generates a larger proton–motive force. The ratio $v_{BD}/(v_{BD} + v_{BO3})$ is shown as a function of growth rate. At low growth rates, only the CYTBO3 reaction is active, while the fluxes of the two reactions are comparable at large growth rates. In both panels we show average fluxes obtained from 1000 independent realizations of the weights w_i for each value of w_C in glucose minimal medium.

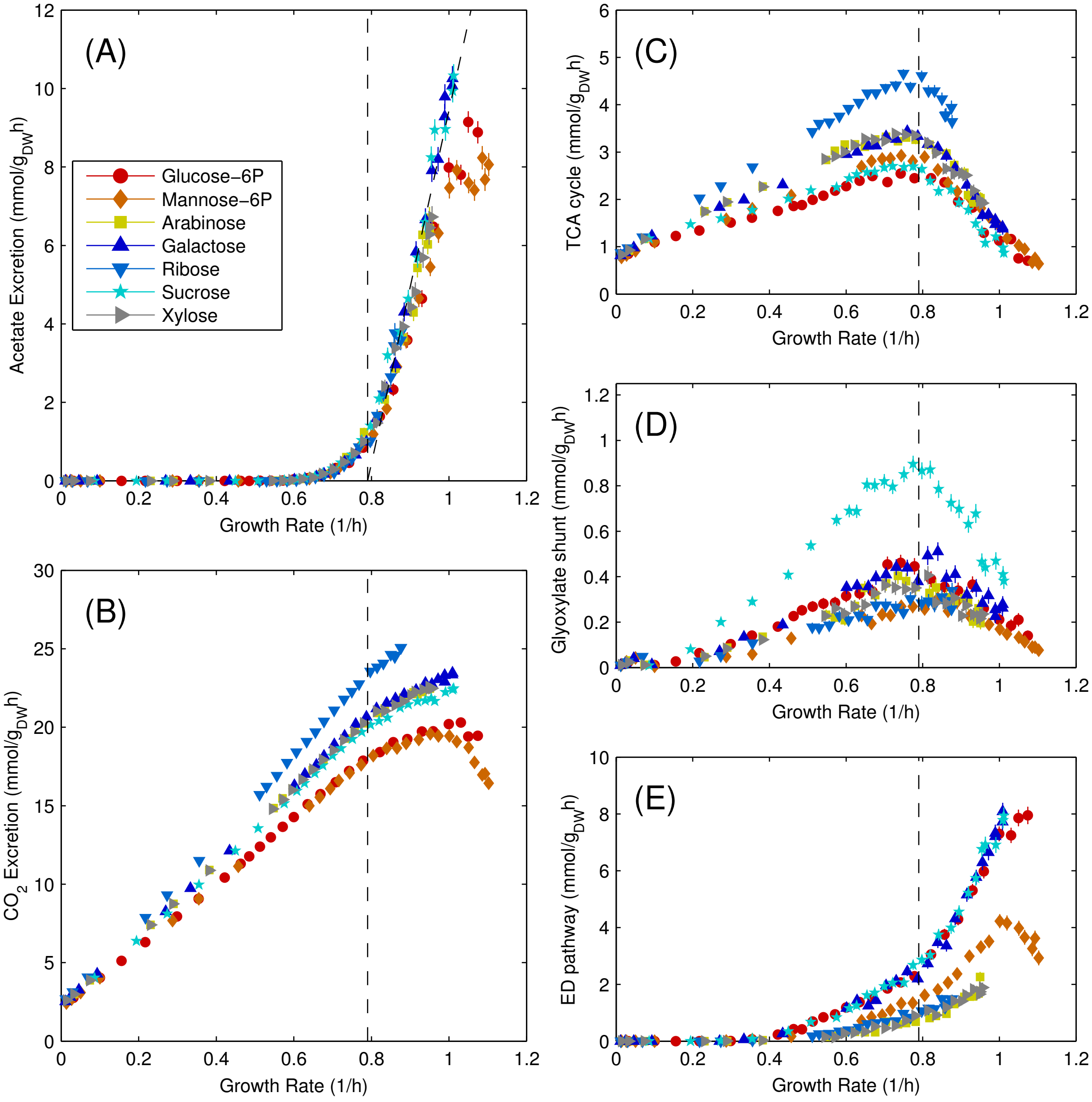

Figure G: CAFBA solutions for seven different glycolitic carbon sources (glucose-6P, mannose-6P, arabinose, galactose, ribose, sucrose, xylose). Acetate excretion rate is consistent for all carbon sources, with slight deviations in the case of phosphorylated carbon sources. The flux through the ED pathway is also heterogeneous, although showing similar trends from carbon to carbon. In all panels the values $\langle w \rangle = 8.8 \times 10^{-4}$ $g_{DW}h/mmol$ and $\delta = 1$ are used. The averages are computed using 500 samples for each value of w_C.

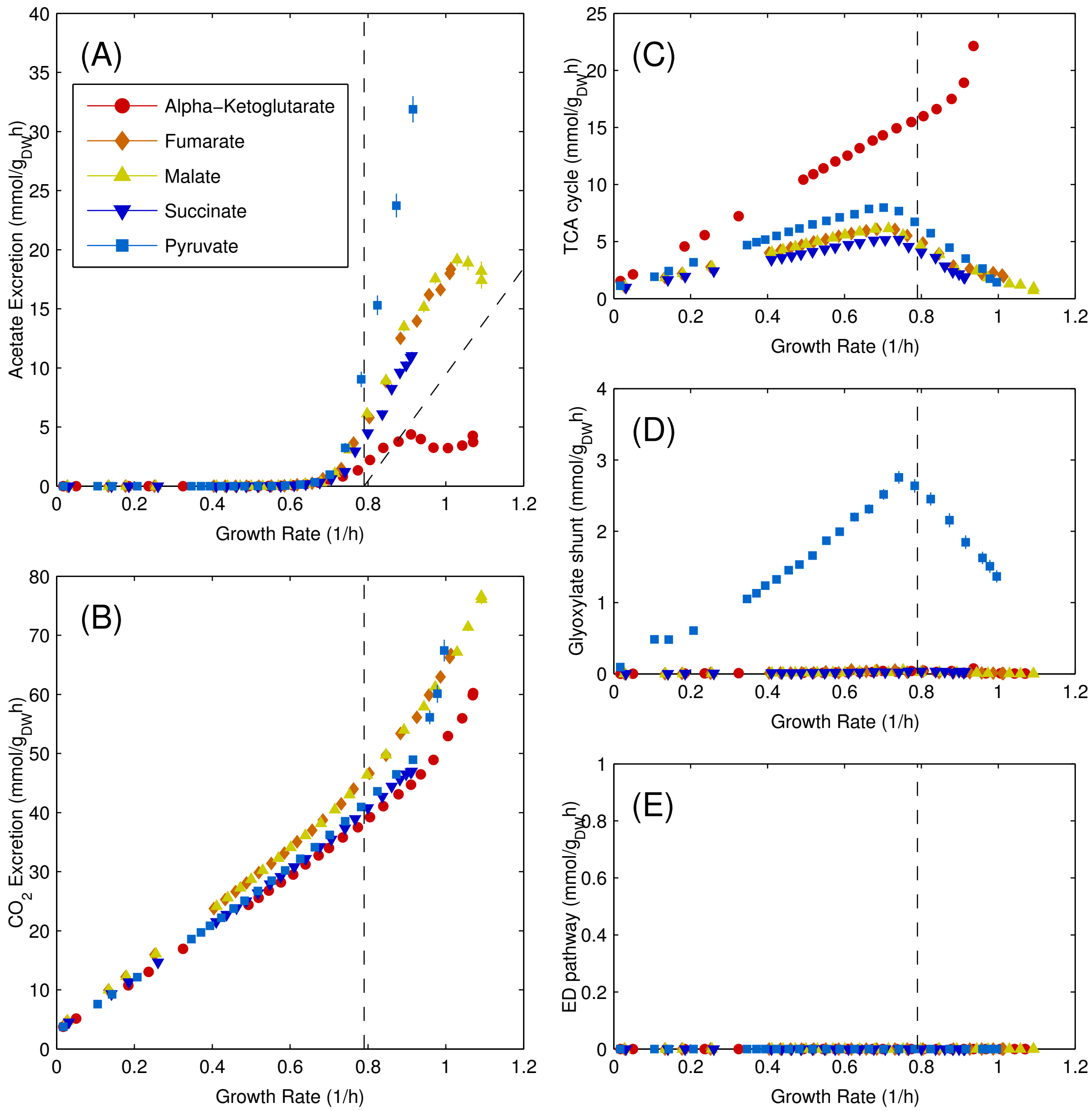

Figure H: CAFBA solutions for five different TCA carbon sources (α-ketoglutarate, fumarate, malate, succinate, pyruvate). Carbon dioxide production is much higher for these non-glycolytic carbon sources, although acetate excretion is still present for growth rates larger than $\lambda_{ac} = 0.79$/h. In all panels the values $\langle w \rangle = 8.8 \times 10^{-4}$ g_{DW}h/mmol and $\delta = 1$ are used. The averages are computed using 500 samples for each value of w_C.

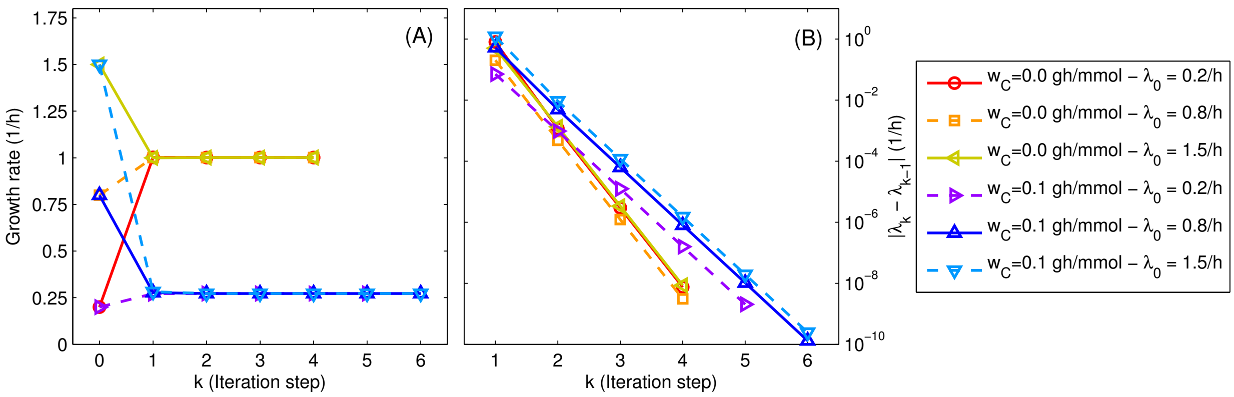

Figure I: Growth-rate dependent biomass composition can be handled in CAFBA (and also in standard FBA) by means of an iterative procedure, in which the growth rate λ_k at step k is obtained by computing the optimal CAFBA solution using the biomass composition obtained at the previous step, $\beta(\lambda_{k-1})$. (A): Growth rate λ_k at each step k of the iterative procedure, for different values of w_C and the initial growth rate guess λ_0. (B): differences between growth rates at two consecutive steps (same as panel A). We stopped the algorithm when $|\lambda_k - \lambda_{k-1}| < 10^{-8}$/h, even if thresholds as large as 10^{-4}/h can be used in practice.

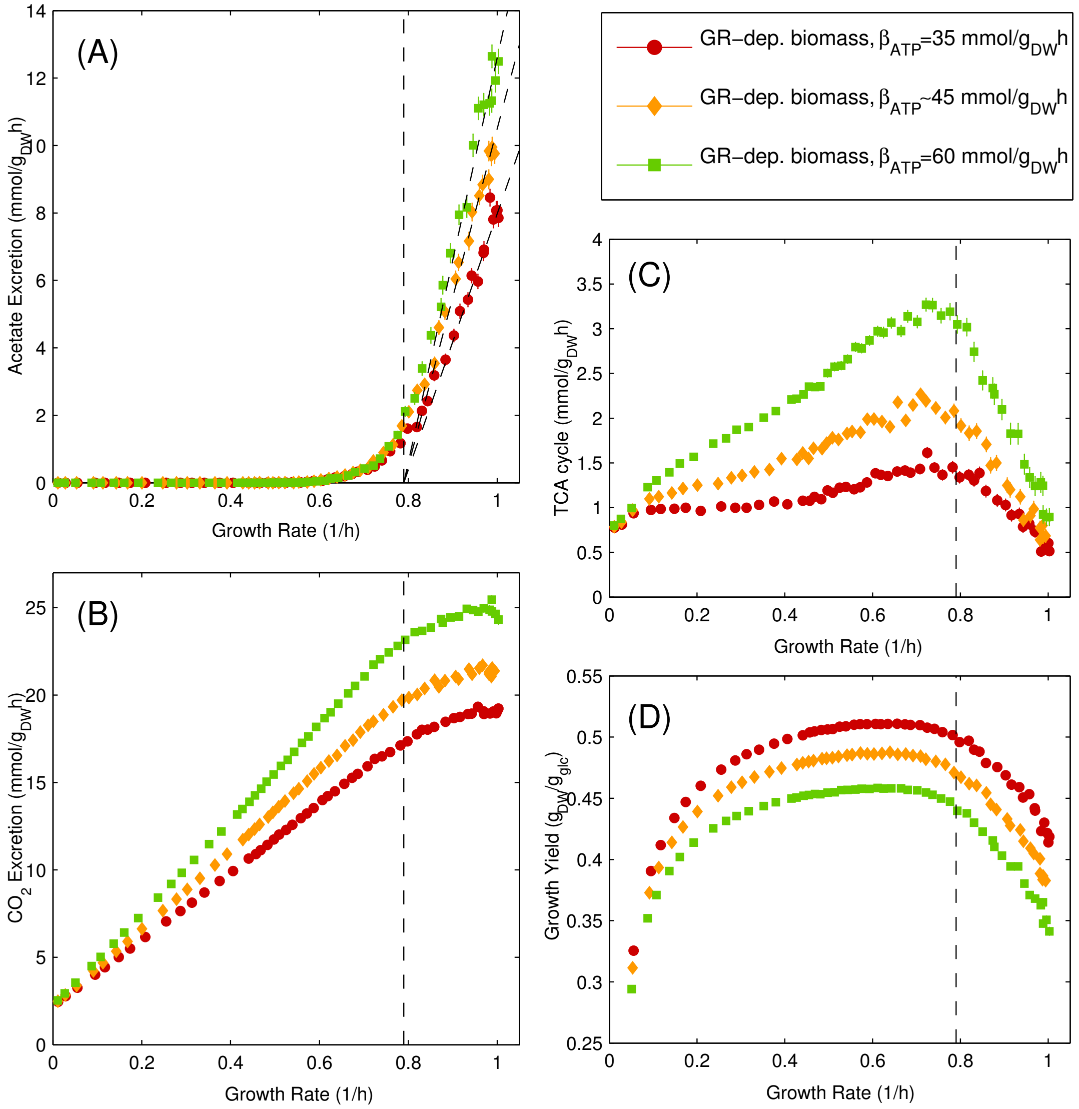

Figure J: As in Fig. 6, we show here fluxes and growth yields obtained by CAFBA in glucose minimal medium with variable biomass composition for three different growth-dependent ATP hydrolysis rates: 35, 45.5608 (as in the *iJR*904 *E. coli* model) and 60 mmol ATP/$\mathrm{g_{DW}}$. The panels show (A) acetate excretion, (B) carbon dioxide excretion, (C) TCA (AKG dehydrogenase) flux, (D) growth yield. In this case, however, the value of $\langle w \rangle$ was tuned as to ensure that the average maximum growth rate is 1/h. This time the slope of acetate excretion flux depends on the growth-rate dependent ATP hydrolysis rate, β_{ATP}, attaining the values 60, 50 or 38 mmol/$\mathrm{g_{DW}}$. However, the x–intercept is independent on β_{ATP}, being $\lambda_{ac} = 0.79$/h in all cases. We used $\langle w \rangle = 9.9 \times 10^{-4}$ $\mathrm{g_{DW}}$h/mmol for $\beta_{ATP} = 35$ $\mathrm{mmol_{ATP}/g_{DW}}$, $\langle w \rangle = 9.2 \times 10^{-4}$ $\mathrm{g_{DW}}$h/mmol for $\beta_{ATP} = 45.5608$ $\mathrm{mmol_{ATP}/g_{DW}}$, and $\langle w \rangle = 8.2 \times 10^{-4}$ $\mathrm{g_{DW}}$h/mmol for $\beta_{ATP} = 60$ $\mathrm{mmol_{ATP}/g_{DW}}$, while in all cases $w_C \geq \langle w \rangle$ and $w_{\max}/w_{\min} = 10$ (i.e. $\delta = 1$).

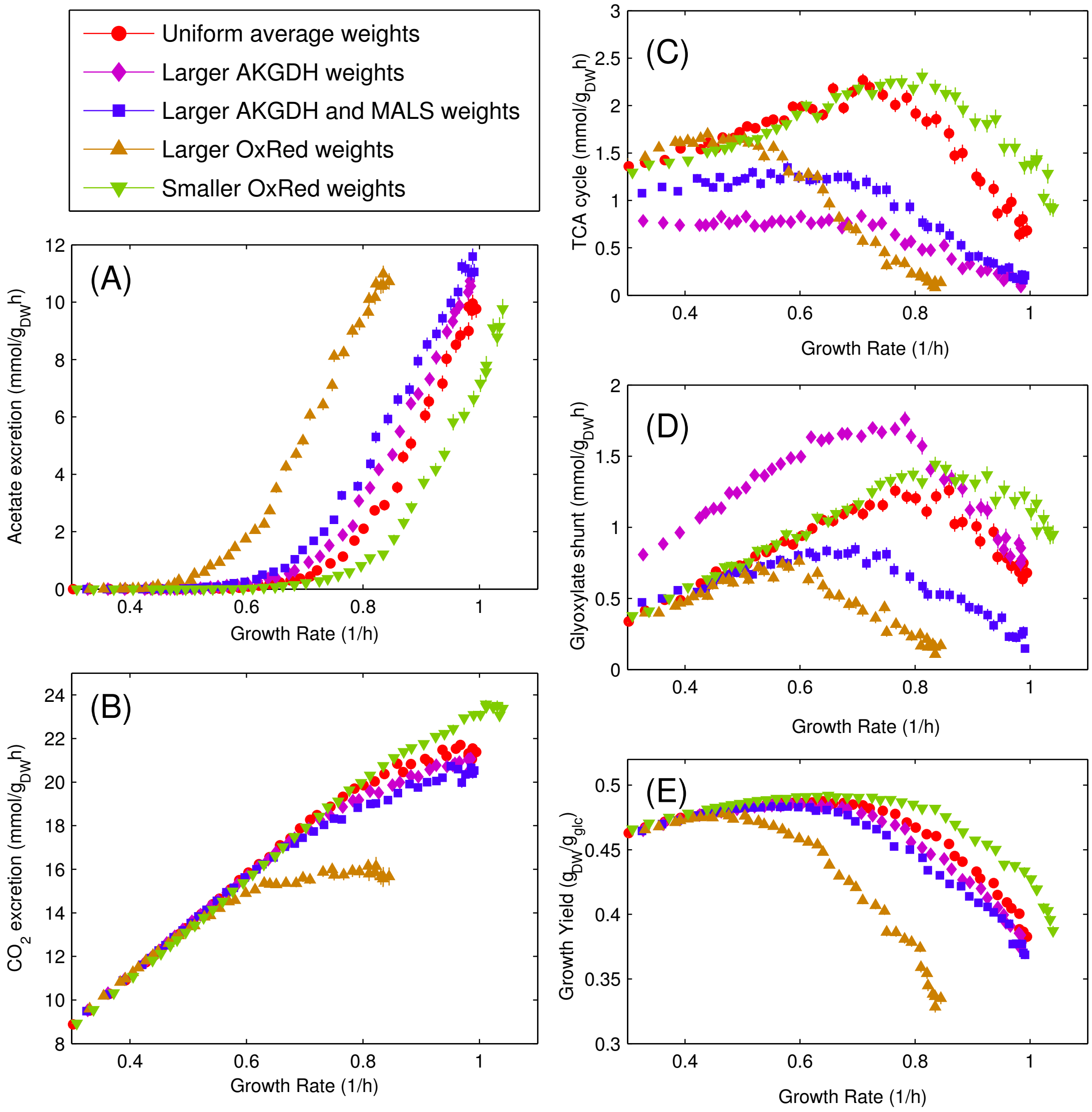

Figure K: Fluxes and growth yields obtained by CAFBA in glucose minimal medium with variable biomass composition for different fine-tunings of single weights. (A) Acetate excretion, (B) carbon dioxide excretion, (C) TCA flux, (D) glyoxylate shunt flux and (E) growth yield. Instead of extracting all weights of the E-sector from the same probability distribution, we used a different average value for single reactions, while keeping the average weight for all other reactions to the value $\langle w \rangle = 9.2 \times 10^{-4}$ $g_{DW}h/mmol$. Red dots (●): all reactions have the same average weight. Purple diamonds (♦): αKG–dehydrogenase weight is multiplied by a factor 5; Blue squares (■): both αKG–dehydrogenase and malate synthase weights have been multiplied by 5; Up–pointing gold triangles (▲): Oxidative phosphorilation reactions (ubiquinol oxidases) weights have been multiplied by 5; Down–pointing green triangles (▼): Oxidative phosphorilation reactions weights have been divided by 5.

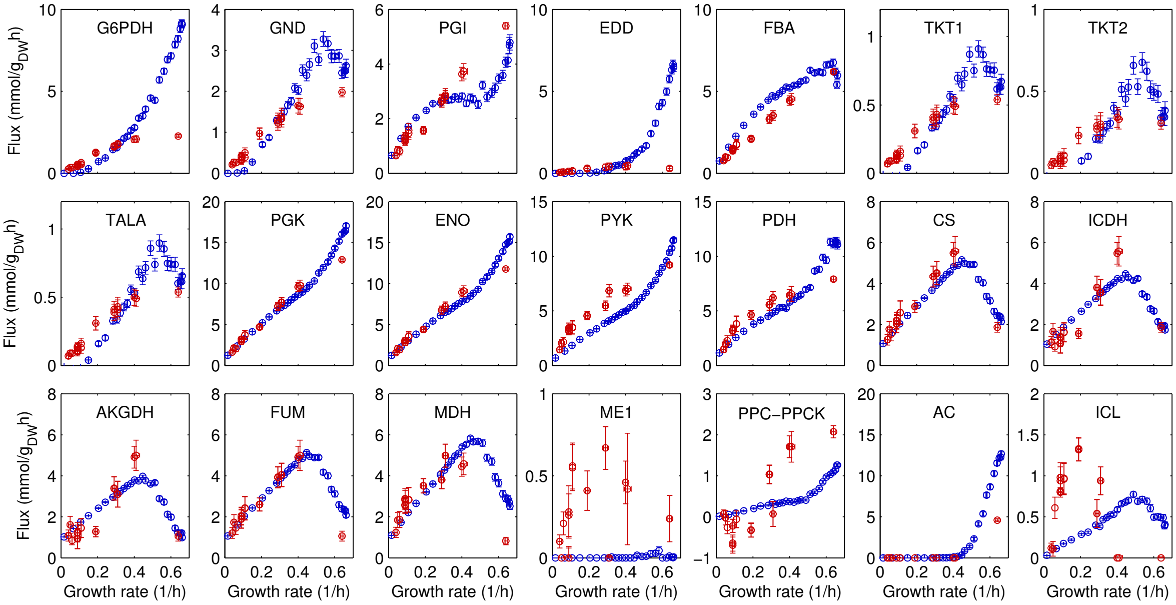

Figure L: Comparison between CAFBA fluxes (blue markers) and experimental fluxes for the *E. coli* MG1655 strain (red markers). All data are derived from chemostat experiments with glucose minimal medium [26] except for the points at $\lambda = 0.64$/h, obtained from batch culture experiments [27]. To match the low growth yield found in [26] (see Fig. 3B therein), we set the ATP maintenance flux at $v_{ATPM} = 9$ mmol ATP/g$_{\mathrm{DW}}$h and the growth-dependent ATP hydrolysis rate at $\beta_{ATP} = 90$ mmol$_{\mathrm{ATP}}$/g$_{\mathrm{DW}}$. Correspondingly, the average weight of the E-sector was set to $\langle w \rangle = 1.15 \cdot 10^{-3}$ g$_{\mathrm{DW}}$h/mmol in order to keep the maximum growth rate close 0.7/h, consistently with data. CAFBA obtains a good agreement for most of the fluxes. The qualitative behaviour of the flux through the glyoxylate shunt is captured, whereas the largest discrepancy is found for the ED pathway (G6PDH and EDD fluxes). Note that the optimal CAFBA flux through this pathway, which is computed assuming growth on a single carbon source, depends strongly on the specific substrate (see Fig. 4 in the Main Text). However, it is unlikely that metabolic fluxes *in vivo* are optimized for growth on a single carbon source.

Supplementary References

1. Benyamini T, Folger O, Ruppin E, Shlomi T (2010) Method flux balance analysis accounting for metabolite dilution. *Genome Biol* 11(4):R43.

2. Boer VM, Crutchfield CA, Bradley PH, Botstein D, Rabinowitz JD (2010) Growth-limiting intracellular metabolites in yeast growing under diverse nutrient limitations. *Mol Biol Cell* 21(1):198–211.

3. Valgepea K, Adamberg K, Seiman A, Vilu R (2013) Escherichia coli achieves faster growth by increasing catalytic and translation rates of proteins. *Mol Biosyst* 9(9):2344–2358.

4. Hui S et al. (2015) Quantitative proteomic analysis reveals a simple strategy of global resource allocation in bacteria. *Molecular systems biology* 11(2).

5. Chubukov V, Gerosa L, Kochanowski K, Sauer U (2014) Coordination of microbial metabolism. *Nature Reviews Microbiology* 12(5):327–340.

6. Ogasawara H, Ishida Y, Yamada K, Yamamoto K, Ishihama A (2007) Pdhr (pyruvate dehydrogenase complex regulator) controls the respiratory electron transport system in escherichia coli. *Journal of bacteriology* 189(15):5534–5541.

7. Beg Q et al. (2007) Intracellular crowding defines the mode and sequence of substrate uptake by Escherichia coli and constrains its metabolic activity. *Proc Natl Acad Sci USA* 104(31):12663–12668.

8. Vazquez A et al. (2008) Impact of the solvent capacity constraint on E. coli metabolism. *BMC Syst Biol* 2(1):7.

9. Lerman JA, Chang RL, Hyduke DR, Palsson BØ, et al. (2013) Genome-scale models of metabolism and gene expression extend and refine growth phenotype prediction. *Mol Syst Biol* 9(1).

10. Vemuri G, Altman E, Sangurdekar D, Khodursky A, Eiteman M (2006) Overflow metabolism in escherichia coli during steady-state growth: transcriptional regulation and effect of the redox ratio. *Applied and environmental microbiology* 72(5):3653–3661.

11. Valgepea K et al. (2010) Systems biology approach reveals that overflow metabolism of acetate in escherichia coli is triggered by carbon catabolite repression of acetyl-coa synthetase. *BMC Syst Biol* 4(1):166.

12. Basan M et al. (2015) Efficient allocation of proteomic resources for energy metabolism results in acetate overflow. *Nature* 528:99–104.

13. Rao N, Torriani A (1990) Molecular aspects of phosphate transport in Escherichia coli. *Molecular Microbiology* 4:1083–1090.

14. Scott M, Gunderson CW, Mateescu EM, Zhang Z, Hwa T (2010) Interdependence of cell growth and gene expression: origins and consequences. *Science* 330(6007):1099–1102.

15. Schellenberger J, Park JO, Conrad TM, Palsson BØ (2010) Bigg: a biochemical genetic and genomic knowledgebase of large scale metabolic reconstructions. *BMC bioinformatics* 11(1):213.

16. You C et al. (2013) Coordination of bacterial proteome with metabolism by cyclic AMP signalling. *Nature* 500(7462):301–306.

17. Monod J (1949) The growth of bacterial cultures. *Annual Reviews in Microbiology* 3(1):371–394.

18. Schaechter M, Maaløe O, Kjeldgaard N (1958) Dependency on medium and temperature of cell size and chemical composition during balanced growth of salmonella typhimurium. *J Gen Microbiol* 19(3):592–606.

19. O'Brien EJ, Lerman JA, Chang RL, Hyduke DR, Palsson BØ (2013) Genome–scale models of metabolism and gene expression extend and refine growth phenotype prediction. *Mol Syst Biol* 9(1).

20. Taymaz-Nikerel H, Borujeni AE, Verheijen PJ, Heijnen JJ, van Gulik WM (2010) Genome-derived minimal metabolic models for escherichia coli mg1655 with estimated in vivo respiratory atp stoichiometry. *Biotechnol Bioeng* 107(2):369–381.

21. Pramanik J, Keasling J (1997) Stoichiometric model of *Escherichia coli* metabolism: incorporation of growth–rate dependent biomass composition and mechanistic energy requirements. *Biotechnology and bioengineering* 56(4):398–421.

22. Reed JL, Vo TD, Schilling CH, Palsson BO, et al. (2003) An expanded genome–scale model of *Escherichia coli* K–12 (*iJR*904 GSM/GPR). *Genome Biol* 4(9):R54.

23. Feist, AM ea (2007) A genome-scale metabolic reconstruction for Escherichia coli K-12 MG1655 that accounts for 1260 ORFs and thermodynamic information. *Mol Syst Biol* 3(1).

24. Orth JD et al. (2011) A comprehensive genome-scale reconstruction of *escherichia coli* metabolism. *Mol Syst Biol* 7(1).

25. Sauer U, Canonaco F, Heri S, Perrenoud A, Fischer E (2004) The soluble and membrane–bound transhydrogenases udha and pntab have divergent functions in nadph metabolism of escherichia coli. *J Biological Chem* 279(8):6613–6619.

26. Nanchen A, Schicker A, Sauer U (2006) Nonlinear dependency of intracellular fluxes on growth rate in miniaturized continuous cultures of Escherichia coli. *Appl Environ Microbiol* 72:1164–1172.

27. Perrenoud A, Sauer U (2005) Impact of global transcriptional regulation by ArcA, ArcB, Cra, Crp, Cya, Fnr, and Mlc on glucose catabolism in Escherichia coli. *Journal of Bacteriology* 187:3171–3179.
