# Supplementary figures and images for: Constrained Allocation Flux Balance Analysis
Source: PLoS Comput Biol. 2016 Jun 29;12(6):e1004913. doi: 10.1371/journal.pcbi.1004913 (PMC4927118; doi:10.1371/journal.pcbi.1004913)

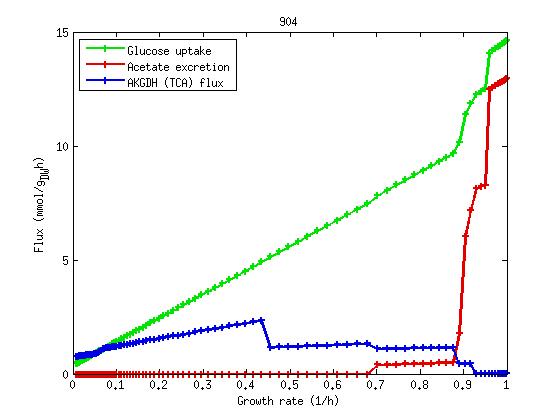

Supplement: S1 Code — COBRA-compatible Matlab functions implementing CAFBA. (ZIP) [file pcbi.1004913.s002.zip › Supplementary Code/carbonLimitation.jpg]
